# Learning Probabilistic Inference through Spike-Timing-Dependent Plasticity^1^,^2^,^3^

**DOI:** 10.1523/ENEURO.0048-15.2016

**Published:** 2016-06-21

**Authors:** Dejan Pecevski, Wolfgang Maass

**Affiliations:** Institute for Theoretical Computer Science, Graz University of Technology, A-8010 Graz, Austria

**Keywords:** network plasticity, neural computation, probabilistic inference, STDP, uncertain information

## Abstract

Numerous experimental data show that the brain is able to extract information from complex, uncertain, and often ambiguous experiences. Furthermore, it can use such learnt information for decision making through probabilistic inference. Several models have been proposed that aim at explaining how probabilistic inference could be performed by networks of neurons in the brain. We propose here a model that can also explain how such neural network could acquire the necessary information for that from examples. We show that spike-timing-dependent plasticity in combination with intrinsic plasticity generates in ensembles of pyramidal cells with lateral inhibition a fundamental building block for that: probabilistic associations between neurons that represent through their firing current values of random variables. Furthermore, by combining such adaptive network motifs in a recursive manner the resulting network is enabled to extract statistical information from complex input streams, and to build an internal model for the distribution p^*^ that generates the examples it receives. This holds even if p^*^ contains higher-order moments. The analysis of this learning process is supported by a rigorous theoretical foundation. Furthermore, we show that the network can use the learnt internal model immediately for prediction, decision making, and other types of probabilistic inference.

## Significance Statement

Most memory models focus on pattern completion as computational operation for memory recall. However, obviously our brains can also answer questions that involve diverse experiences in unexpected ways. Consider for example the question whether we went with Mr. X more often to lunch than to dinner, or whether Mr. X would probably like the new restaurant Z. These queries could not be anticipated during the formation of memory traces. Still we can answer such queries (which are special cases of probabilistic inference: estimation of a posterior marginal) without any effort. We propose a model based on experimentally observed rules for synaptic plasticity (STDP), which explains how a network of spiking neurons can acquire this capability.

## Introduction

A large number of experimental data from neuroscience and cognitive science suggest that the brain performs probabilistic inference for a large number of probability distributions p^*^ that describe different aspects of the environment of the organism and its interaction with the environment (Griffiths and Tenenbaum, 2006; Bonawitz et al., 2014). In models for probabilistic inference in networks of neurons one has so far (with the exception of Siegelmann and Holzman, 2010; see Discussion) programmed the underlying distribution p^*^ into the architecture and parameters of the network model. However brains have to learn internal models *p* of salient distributions p^*^ in their environment from examples that are generated by p^*^. We present in this article the first model that explains how networks of spiking neurons could learn such internal models *p* through experimentally supported local plasticity rules. Furthermore, we show how a network of spiking neurons can store the learnt information in a way that makes it readily accessible for probabilistic inference.

Two different approaches how networks of neurons in the brain could execute probabilistic inference have been proposed. One approach emulates an arithmetical (deterministic) algorithmic method for performing probabilistic inference through a suitable distributed organization of sums and products of probabilities (referred to as belief propagation or message passing; Steimer et al., 2009). The other approach for emulating probabilistic inference in networks of spiking neurons is based on the assumption that a network of neurons can “embody” a distribution *p* in such a way that it can generate samples from *p*. Probabilistic inference for *p* can then be performed through simple operations on these samples, for example the computation of a posterior marginal just requires to look at the distribution of the random variable of interest in these samples. This approach is known in computer science as Markov chain Monte Carlo (MCMC) sampling, and is widely used to perform probabilistic inference also for complex distributions *p* for which belief propagation approaches have no guarantee to provide correct answers. Whereas the previous approach prefers a deterministic network, where every stochasticity is detrimental for the performance, this second approach requires an inherently stochastic network of spiking neurons (Buesing et al., 2011). It has been argued that the dynamics of networks of neurons in the brain is in fact highly stochastic, both on the basis of inherently stochastic features of its components (such as stochastic synaptic release), and on the basis of trial-to-trial variability in neural recordings and observed variability in behavioral outputs.

The learning model that is presented in this article ties in to this second approach, and shows that stochastic networks of neurons are able to automatically absorb the relevant statistical information from examples that they receive. As a result, we have now one first complete theory for the emergence of probabilistic inference in networks of spiking neurons through learning. We focus in this article on somewhat idealized models for spiking neurons and plasticity rules, which allow us to give rigorous proofs for the convergence of learning.

We first show how an extension of an ubiquitous network motif of cortical microcircuits, interconnected populations of pyramidal cells with lateral inhibition (Winner-Take-All (WTA) circuits; Douglas and Martin, 2004; Nessler et al., 2013), gives rise to the basic building block for absorbing probabilistic information from examples. The output neurons of an array of WTA-circuits form the hidden layer of a three-layer feedforward learning module, to which we refer as a “stochastic association module.” We show that such a module can learn through spike-timing-dependent plasticity (STDP) and plasticity of the excitability of neurons (intrinsic plasticity) stochastic associations between the random variables that are encoded by the firing of neurons on its first and third layers. The module can learn this probabilistic information from the statistics of activation and coactivation of these neurons when the network processes examples that are provided by its environment. An important second finding is that recursive combinations of this network module can learn even complex probabilistic relationships between large numbers of random variables. This network learning capability is in fact universal in the sense that the underlying theory implies a proof of principle that an approximation to any distribution p^*^ over discrete random variables can be learnt by exposing the network to examples drawn from p^*^. In fact, one can show that if the network is too small or has too few connections for learning a close approximation of p^*^, it will still strive toward approximating p^*^ as well as it can, given its limited resources. The understanding of this learning process is supported by the theory of Expectation Maximization (EM).

## Results

Previous models for probabilistic inference in networks of spiking neurons have shown that one can program the parameters (eg, conditioned probability tables) of a given distribution p^*^ over discrete random variables into a network of idealized models for spiking neurons, provided that the network has a suitable architecture. We provide in this article a proof of principle that these parameters of p^*^ do not have to be programmed into the network: they can be learnt by a network N of spiking neurons via simple local plasticity rules from examples $\overset{˜}{y}$ that are generated by p^*^. This does not hold for every neural network N, but like in any existing model for probabilistic inference in neural networks, only under suitable assumptions about the architecture of N. This result provides a proof of principle that networks of neurons in the brain can not only perform probabilistic inference for distributions p^*^ whose parameters are specified in the genetic code, but also for distributions p^*^ that an organism encounters in its environment.

The underlying theory of EM does not guarantee that p^*^ can be learnt perfectly. However it implies that a network N with a suitable architecture is expected to make progress in creating an increasingly more accurate internal model *p* for p^*^ when it receives more and more examples that are generated by p^*^. EM does not guarantee that the internal model *p* converges to p^*^, but it implies that the network learning process cannot “run around in circles” where *p* moves forth and back between better and worse approximations of p^*^. This learning result is general insofar as it shows that internal models *p* can be learnt by a network N with a suitable architecture for external distributions p^*^ over any number of discrete random variables, with arbitrary, also higher-order, dependencies among these random variables. However, although the architecture of N will obviously have to depend on the number of random variables of p^*^, we show that it suffices to assume that it consists of recursive interconnections of different copies of a simple generic network motif, to which we refer as a stochastic association module. This network motif is a three-layer feedforward network of excitatory spiking neurons with lateral inhibition on the hidden layer (see Fig. 2). We show that this simple microcircuit motif can be viewed as an atomic learning module, that extracts via STDP and intrinsic plasticity from examples probabilistic associations between input variables **x** and output variable *z* that are encoded through population coding on its input and output layer. An example is presented in Figure 3. We will then show in the following subsection that this atomic learning module can be recursively combined to form a network that automatically approximates through STDP and intrinsic plasticity arbitrarily complex distributions p^*^ over many discrete random variables from examples generated by p^*^. In other words, this network learns an internal probabilistic model *p* for its stochastic environment p^*^. Furthermore, this network has the property that it can readily apply this internal model by carrying out probabilistic inference for *p* through its inherent stochastic dynamics. Examples are presented in Figures 6–9.

The neurons in our models are stochastic integrate-and-fire neurons, which have been shown to match biological data quite well (Jolivet et al., 2006; Mensi et al., 2011; Gerstner et al., 2014). We assume that a neuron has at any time *t* the instantaneous firing probability density $\rho (t)=\frac{1}{\tau }\exp\;(u(t))$, where *u*(*t*) is its membrane potential and *τ* is a time constant. When it fires a spike, the neuron enters an absolute refractory period of duration *τ* after which it resumes its stochastic firing. The membrane potential $u(t)=\sum_{i}w_{i}\epsilon_{i}(t)+b$ is assumed to be the sum of the PSPs $\epsilon_{i}(t)$ elicited by the spikes from its presynaptic neurons, where *w_i_* is the synaptic efficacy of the *i*-th synapse (and *b* is the bias of the neuron). The theoretically best tractable shape of a PSP $\epsilon_{i}(t)$ would be a step function of length *τ*. However, we show in Examples 1 and 2 that the relevant learning properties also hold for α-shaped EPSPs that are commonly considered in theoretical neuroscience. On the side, we would like to point out that for biological neurons the EPSPs vary from shapes with a pronounced initial peak to shapes with smooth hills in dependence of the distance of the synapse to the soma (Williams and Stuart, 2002), and obtain yet other shapes if amplified through NMDA or Ca spikes (Larkum et al., 2009).

We use a simple STDP rule, which has the advantage of being theoretically tractable. Let *w* be the weight of the synapse at the connection from some presynaptic neuron *ν_pre_* to a postsynaptic neuron *ν_post_*. At each postsynaptic spike of neuron *ν^post^* at time *t* this weight undergoes an update: w←w+ηΔw, where *η* is the learning rate and

(1) $$\Delta w=\{\begin{array}{cc} e^{-(w+w_{-})}-1, & \text{if}\;\nu_{pre}\;\text{fired in}\;[t-\tau ,t]\;\;,\\ -1, & \text{if}\;\nu_{pre}\;\text{did not fire in}\;[t-\tau ,t]\;. \end{array}$$

**Figure 1.**
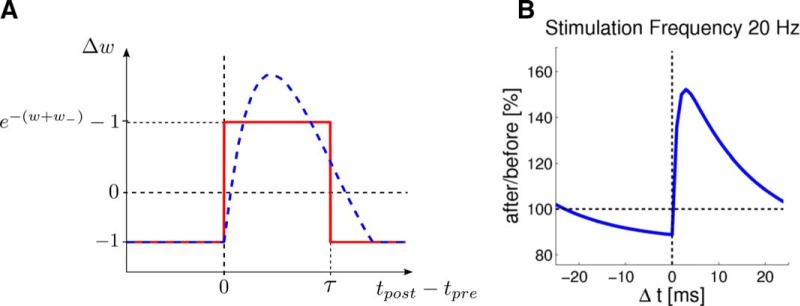
STDP curves of the synaptic plasticity rule. ***A***, The STDP curves show the weight change for a presynaptic spike at time *t_pre_* and a postsynaptic spike at time *t_post_*, for different time differences *t_post_* – *t_pre_*. The red curve represents STDP for the simple rule that is used in the theoretical derivations. In computer simulations, we used also an STDP rule shown with the blue curve, that has a smoother, more biologically realistic shape. ***B***, The change of the synaptic efficacy after a stimulation protocol where both the presynaptic and postsynaptic neuron fire at a frequency of 20 Hz, for different time differences Δ*t* between a postsynaptic and presynaptic spike. The STDP curve shifts more toward LTP, and depression is no longer time independent due to overlapping PSPs (Nessler et al., 2013, their Fig. 4). This STDP curve is quite similar to experimental data (Sjöström et al., 2001).

The parameter *w*_–_ is a baseline parameter in the learning rule, and *τ* is a parameter that corresponds to the duration of postsynaptic potentials (PSPs). Figure 1*A* shows the resulting STDP curve. The rule exhibits LTP only for pre-before-post spiking within a time window of duration *τ*, otherwise it exhibits LTD. The causal part of the STDP window curve has the same shape as the PSP kernel, which is similar to other theoretically derived plasticity rules (Toyoizumi et al., 2005; Pfister et al., 2006). The properties of this plasticity rule were studied by Nessler et al. (2013). It was shown there that it supports learning of an internal probabilistic model of the inputs in a WTA network. It was also shown there that the weight dependence of Δ*w* in Equation 1 fits quite well to experimental data. Figure 1*B* shows that the shape of the STDP curve according to Equation 1 looks like the commonly considered one when applying an intermediate pairing frequency of 20 Hz (Sjöström et al., 2001 shows experimental data on the dependence of the shape of the STDP curve on the pairing frequency).

It is well known that the excitability of neurons changes in dependence of their history of firing activity (Daoudal and Debanne, 2003; Cudmore and Turrigiano, 2004). We model this intrinsic plasticity of neurons through a simple rule according to which the bias *b* of a neuron changes at each spike of the neuron according to b←b+η′ Δb, with:

(2) $$\Delta b=\tau {\;e}^{-(b+b_{-})},$$

where η′ is a learning rate and *b*_–_ is a baseline parameter. In addition, we assume that the bias exhibits constant decay according to the differential equation:

(3) $$\frac{db}{dt}=-\eta \prime .$$

### A network module for learning stochastic associations

The atomic learning module in our model is a simple microcircuit motif that learns associations between some array of random variables $x=(x^{1},. . .,x^{I})$ and another random variable *z* from examples 〈x,z〉 that are presented to the network. The variables **x** and *z* could for example represent different higher-level features of an image. Or the variables **x** could represent higher level features of some visual stimulus, and the variable *z* a feature of a simultaneously occurring auditory stimulus. More formally, we assume that the network is exposed to examples 〈x,z〉 consisting of concrete assignments of discrete values to the variables **x** and *z*, that are drawn from some unknown distribution p^*^(**x**, *z*). We want to determine under what conditions a network module is able to create autonomously from exposure to these examples an internal model *p*(**x**, *z*) for p^*^(**x**, *z*), that approximates p^*^ when the number of examples grows. Note that in general the same input **x** will occur in combination with different values *z*(1), *z*(2),  . . .  of *z* in the training examples, and the goal of learning is to learn for each value *z*(*i*) the probability that it occurs for input **x**. Hence, the learning performance will not be evaluated by counting errors, ie, deviations from a target output value. Rather, it will be evaluated by how well the network reproduces for any input value **x** the distribution of output values *z*.

**Figure 2.**
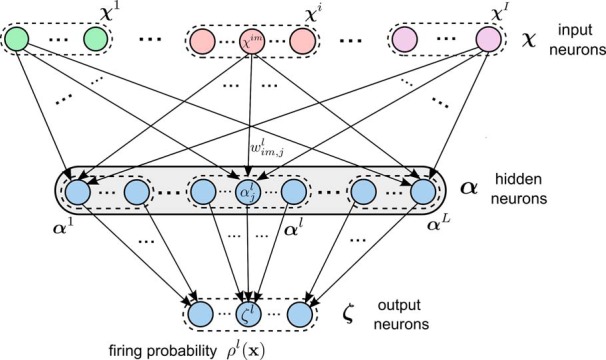
Structure of a stochastic association module that is able to learn probabilistic associations between multinomial variables $x=(x^{1},. . .,x^{I})$ and *z* through STDP. Populations of neurons $\chi^{i}$ (i=1, . . . ,I) on the first layer encode the values of input variables *x^i^*. The population of neurons **ζ** on the third layer encodes the value of *z*. The hidden layer consists of populations of excitatory neurons $\alpha^{l}$ (*I* = 1, . . . , *L*) that are subject to lateral inhibition. STDP applied to the weights $w_{im,j}^{l}$ of synaptic connections from the first layer to the neurons α on the hidden layer enables the network to approximate for any network input **x** through the firing probability of neurons on the third layer the distribution of values *z* that were associated with **x** in previously processed examples 〈x,z〉.

We show that a three-layer network of spiking neurons with the architecture shown in Figure 2 can accomplish this learning task through STDP on synaptic connections from the first to the second layer and intrinsic plasticity of excitatory “hidden” neurons α on the second layer. The weights of synaptic connections between the second and third layers are assumed to be fixed. These weights are assumed to have a large value, so that the firing of a neuron *α* on the second layer causes with very high probability the firing of the neuron on layer 3 to which it is connected.

We assume that each of the random variables **x** and *z* is represented by a population of neurons (“population coding”), with each value of the variable encoded by a separate neuron in the population, as indicated at the top of Figure 2 for the variables *x^i^*, and at the bottom for the variable *z*. The firing of a particular neuron *χ^im^* in the population coding of variable *x^i^* encodes the fact that *x^i^* assumes the value *m* in the currently presented example. Similarly the firing of neuron *ζ^l^* in the population ζ for the variable *z* encodes the value *l* of this variable (Fig. 2). Finally, we assume that an example 〈x,z〉 is presented to this three-layer network during learning in the following manner: Those neurons in the first layer that represent the given values of the variables **x** are made to fire at a high rate, whereas the other neurons in the first layer are inhibited and kept silent. In addition, if variable *z* has value *l* in this example, all hidden neurons outside the corresponding subpopulation $\alpha^{l}$ are inhibited, so that they cannot fire (actually, it would suffice to block STDP for these neurons). An alternative view is that only a selected subset of neurons is disinhibited. New experimental data (for review, see Caroni, 2015) suggest that in fact inhibition of synaptic plasticity (especially via Somatostatin-positive neurons) and disinhibition via VIP interneurons (Letzkus et al., 2015) play an important role in the control of plasticity in cortical microcircuits.

When the network does not receive examples, the neurons in this network module fire according to the stochastic dynamics of the model, and no plasticity is assumed to occur. We present in Materials and Methods a rigorous proof that after learning the distribution of output values *z* for a given network input **x** approximates in this stochastic association module the conditional distribution $p^{*}(z|x)$ of the joint distribution $p^{*}(x,z)$ from which the values of *z* and **x** are drawn in the training examples. In fact, one can view this module from a theoretical perspective as an implicit generative model p(x,z; θ) for the examples 〈x,z〉, and one can prove that the network module performs a stochastic search (stochastic online Expectation Maximization) that strives to minimize the Kullback–Leibler divergence $D_{KL}(p^{*}(x,z)\|p(x,z;\theta ))$ between the external distribution p^*^ from which the examples are drawn and its internal model p(x,z; θ) (see Materials and Methods, Theorem 1 and Theorem 1^*^). The implicit generative model of the module is encoded in the synaptic weights and biases of the α neurons.

Because the learning module represents the full joint distribution p(x,z; θ), not just the conditional distribution p(z|x; θ), it is the joint distribution that is considered to be the internal model of the learning module. This is more than just representing p(z|x; θ) as the module also represents the distribution p(x; θ). The distribution p(x; θ) is represented in a sense that all probability values p(x; θ) for each value of **x** can be calculated from the synaptic weights and biases of the α neurons. This can be done by first calculating p(x,z; θ) for each value of *z* based on the probabilistic model (see Materials and Methods), and then marginalizing out *z*. Another reason why p(x,z; θ) is considered as internal model is that the learning rules are based on minimizing the Kullback–Leibler divergence between the internally represented joint distribution p(x,z; θ) and the target distribution *p* ^*^(**x**, *z*) of the examples. In other words, the module implements generative model learning. The conditional distribution becomes important after learning, when the module performs its function realized through the firing of the output neurons that approximates p^*^(z|x). This functional property of the learning module enables composing networks of modules that can learn larger distributions, as described in the section “Recursive combinations of the basic learning module enable efficient learning of complex distributions from examples.”

**Figure 3.**
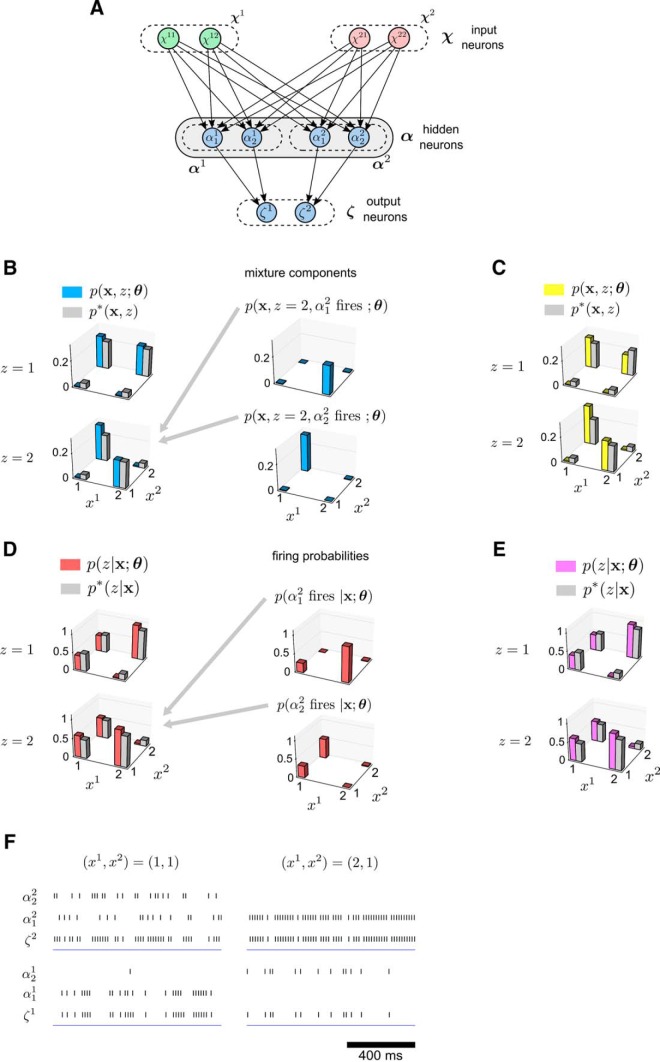
Learning results for Example 1. ***A*,** Structure of the learning module. There are two subpopulations $\alpha^{1},\alpha^{2}$ of hidden neurons that both receive inputs from the two populations on layer 1 that encode the input variables *x* ^1^ and *x* ^2^. Each subpopulation of hidden neurons projects to a different neuron in the population coding of the variable *z* on layer 3. ***B***, Left, The target probability distribution $p^{*}(x,z)$ of the examples (grey bars) and the internal model p(x,z; θ) (blue bars) that is extracted from the examples by the hidden neurons. The learned probabilities match the target probabilities quite well. Right, The two mixture components $p(x,z=2,\alpha_{1}^{2}\text{ fires}\;;\theta )$ and $p(x,z=2,\alpha_{2}^{2}\text{ fires}\;;\theta )$ represented by the hidden neurons $\alpha_{1}^{2}$ and $\alpha_{2}^{2}$ in the subpopulation $\alpha^{2}$. Each has specialized to represent one of the two modes. The resulting internal model p(x,z=2; θ) is a sum of these two mixture components. ***C***, Same as the plots on the left in ***B***, but for a larger network where four hidden neurons were used in each subpopulation $\alpha^{1},\alpha^{2}$ of hidden neurons. This larger size of the subpopulations is suggested by the network construction from Pecevski et al. (2011), because the vector **x** can assume four different values. However, a comparison with ***C*** shows that smaller subpopulations suffice here for good learning performance. ***D***, Left, The target probabilities $p^{*}(z|x)$ (grey bars) compared with the learned firing probabilities of the output neurons $\zeta^{1}$ and *ζ* ^2^ that represent p(z=1|x; θ) and p(z=2|x; θ) respectively. Right, The probability of firing in response to different inputs **x** for the two hidden neurons $\alpha_{1}^{2}$ and $\alpha_{2}^{2}$ that drive $\zeta^{2}$ to fire. ***E***, Same as the left plots in ***D***, but for the larger network as in ***C***. Again, one sees that fewer hidden neurons are needed here than in the construction of Pecevski et al. (2011). ***F***, Firing activity of the hidden neurons and output neurons in the module in response to two different input patterns $\left(x^{1},x^{2})=(1,1\right)$ and $\left(x^{1},x^{2})=(2,1\right)$. The firing rates of $\alpha_{1}^{2}$ and $\alpha_{2}^{2}$ correspond to their probabilities of firing shown in ***D***.

One may wonder why a two-layer network would not suffice for learning such stochastic associations between random variables **x** and *z*. The simplest approach would be a model without hidden neurons, where the strengths of the synaptic connections between the neurons in the population codes for **x** and *z* encode the probability that a vector **x** is encountered in conjunction with a particular value of *z*. But this approach would restrict very much the types of internal models *p*(**x**, *z*) that the network could learn. In particular, it could not handle a situation where the distribution *p* ^*^(**x**, *z* = *l*) is multimodal, ie, when there are multiple modes in the distribution of **x** that are likely to occur in conjunction with a specific value *l* of *z*. For example in Figure 3*B* for *z* = 2, the distribution *p* ^*^(**x**, *z* = 2) has two modes, ie, *x* ^1^ = 1 can occur in combination with *x* ^2^ = 2, and *x* ^1^ = 2 in combination with *x* ^2^ = 1 (whereas the assignments where *x* ^1^ = *x* ^2^ do not occur). The reason for this restriction to unimodal distributions is that the neuron *ζ^l^* that represents *z* = *l* in the population code for *z* would have to represent through the implicit generative model that is defined by the weights of afferent synapses and its excitability the marginal distribution *p* ^*^(**x**, *z* = *l*). However, a single linear neuron can only represent one mode of a probability distribution of **x**. However if one considers more complex neuron models with nonlinear dendritic processing, they can in principle also represent multimodal distributions (Pecevski et al., 2011, their Figs. 4 and 5; Legenstein and Maass, 2011). Hence, with such more complex neuron models a more shallow learning network could potentially be used as a learning module in our architecture.

The three-layer circuit in Figure 2 can be viewed as a minimal model for allowing multimodal distributions of **x** to be associated with a value of *z*. In fact, if one allows sufficiently many hidden neurons α, this representation becomes arbitrarily precise. These hidden neurons α represent combinations of features represented through the neurons $\chi^{i}$ that encode the variables **x**. This mixed coding is reminiscent of experimental data on neurons in the cortex (Rigotti et al., 2013; Mante et al., 2013).

We exploit here a generic property of STDP in WTA circuits, that was made explicit by Nessler et al. (2013) and Habenschuss et al. (2013b): if the neurons in the populations for the variables *x^i^* are synaptically connected to a set of neurons α in a WTA circuit, and these synaptic connections are subject to STDP, then the neurons α learn automatically a multimodal internal model for the distribution of the variables **x**. The learned probabilistic model is a mixture of multinomials. More precisely, each WTA neuron *α* specializes to fire in response to input patterns from one mode of p^*^(**x**). This specialization is produced by the plasticity rules (Eqs. 1, 2), which, when a neuron fires in response to some input pattern, adapt the weights and biases of the neuron so that in the future it fires with higher probability in response to the same pattern. At the same time, the competition enforced by the lateral inhibition between the α neurons tends to prevent that multiple WTA neurons specialize on the same mode of p^*^(**x**).

This emergent property of STDP in WTA circuits was considered by Nessler et al. (2013) and Habenschuss et al. (2013b) in a setting where no association of **x** with other variables *z* needed to be learnt. In order to learn associations with *z*, we apply this mechanism in parallel for every possible value of *z*. In particular, we assume that in the population α of WTA neurons there are disjoint subpopulations $\alpha^{l}$ for each possible value *l* of *z*. The subpopulation $\alpha^{l}$ projects to the *ζ^l^* neuron with strong synaptic weights so that a spike of a neuron in $\alpha^{l}$ causes also the neuron *ζ^l^* to fire (Fig. 2). As the WTA subcircuit $\alpha^{l}$ is allowed to fire only for examples from p^*^(x|z = *l*) it learns to approximate this distribution.

Intrinsic plasticity of the excitability of the hidden neurons $\alpha^{l}$ is also essential for successful learning. As they are not firing during a presentation of example 〈x,z〉 with *z*≠*l*, the only adaptation in them for such example is a decay of their intrinsic excitabilities (Eq. 3). This supports learning of a representation of the marginal probability p^*^(*z* = *l*) in the biases of the neurons in $\alpha^{l}$. Hence, the population $\alpha^{l}$ learns a probabilistic model p(x,z=l; θ) of the target probability distribution p^*^(**x**, *z* = *l*) = p^*^(x|z = *l*) p^*^(*z* = *l*). In this way all populations $\alpha^{l}$ together learn a generative model p(x,z; θ) for the joint distribution $p^{*}(x,z)$ of the presented examples.

Further details can be found in Materials and Methods, section “Theoretical properties of the basic learning module (stochastic association module) and its plasticity.”

### Example 1: the learning module extracts complex stochastic associations from examples

We illustrate the inner workings of the learning module in an example where the task is to learn an internal model of an example target distribution p^*^(*x* ^1^, *x* ^2^, *z*) over binary RVs. This learning task is nontrivial since the distributions of values of **x** that are stochastically associated with the values *z* = 2 and *z* = 1 according to p^*^ are multimodal (Fig. 3*B*, gray bars). After learning, the output neurons of the module should fire for input **x** according to the conditional probability p^*^(z|x). The structure of the network module is depicted in Figure 3*A*. It has two hidden neurons in the populations $\alpha^{1}$ and $\alpha^{2}$, which learn the two modes of p^*^(**x**, *z* = 1) and the two modes of p^*^(**x**, *z* = 2), respectively (Fig. 3*B*). The learning period of the module lasted 1200 s of simulated biological time. During learning, examples from p^*^(**x**, *z*) were presented to the module for 100 ms each. After learning, each WTA subcircuit $\alpha^{l}$ had in fact acquired an approximation of the distribution p^*^(**x**, *z* = *l*), as can be seen in Figure 3*B*. The learning of the internal model in the WTA subcircuit $\alpha^{2}$ is achieved through a process where each hidden neuron specializes to represent one of the two modes of p^*^(**x**, *z* = 2) that are shown in Figure 3*B*. For the subcircuit $\alpha^{1}$ the results are similar (not shown). The learning of an approximation to p^*^(**x**, *z*) as an internal model automatically produces an approximation of the conditional p^*^(z|x) by the firing probabilities of the output neurons (Fig. 3*D*). A typical resulting firing pattern is shown in Figure 3*F*.

### Recursive combinations of the basic learning module enable efficient learning of complex distributions from examples

**Figure 4.**
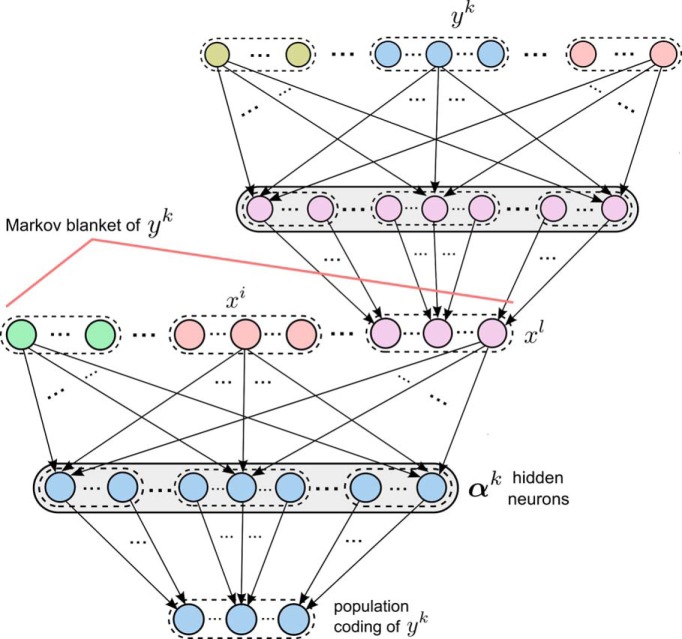
Recursive combination of learning modules. The learning module for the RV *y^k^* at the bottom has the same structure as the modules shown in Figures 2 and 3. For learning complex distributions p^*^ its input variables $x^{1},\text{ . . . },x^{l}$ form a Markov blanket of *y^k^*. Each variable *x^i^* is encoded by the same population coding as the output variables of learning modules, and can therefore be produced by the output of another learning module (as shown for the RV *x^l^*). As here *y^k^* is in the Markov blanket of *x^l^*, *y^k^* appears among the input variables of the upper module, and its corresponding input neurons are the same as the output neurons of the lower module.

The stochastic association module shown in Figures 2 and 3 is self-consistent in the sense that the input variables *x^i^* are encoded through population coding in the same way as the output variable *z*. Hence, one can recursively combine these modules so that the output population of one module becomes part of the input population of another module (Fig. 4). The resulting more complex network is then not only able to learn a single probabilistic association between RVs, but many such associations simultaneously. The basic learning modules form here not only chain connections, but typically also cycles, where the RV that is the output of the second module is simultaneously an input to the first module in the chain (like the variable *y^k^* in Fig. 4).

Elementary results from probability theory imply that such recursive combinations of probabilistic associations between RVs have a very powerful, in fact universal, representation capability: the dependency structure of every probability distribution p^*^ over discrete RVs can be represented as a network of probabilistic associations between each of the RVs *y^k^* and a subset of the other RVs (Bishop, 2006). More precisely, in the representation of an arbitrary distribution p^*^ one has a subnetwork (module) for each RV *y^k^* of p^*^ that has *y^k^* as output variable and the random variables in the Markov blanket **y***^B^* ^(^*^k^* ^)^ of *y^k^* as input variables. The Markov blanket defines a set of random variables so that conditioned on their values, *y^k^* becomes independent from all remaining variables. For example, if p^*^ can be represented by a Bayesian network, it suffices to include in **y***^B^* ^(^*^k^* ^)^ the parents of *y^k^* together with the children of *y^k^*, and their coparents.

Whereas the classical results from probability theory only imply that one can represent any distribution p^*^ over discrete RVs as such recursive network of probabilistic associations, it was shown in (Buesing et al., 2011; Pecevski et al., 2011) that any such target distribution p^*^ can also be represented as stationary distribution of a network of spiking neurons, if suitable parameters (weights and biases) are programmed into the network. One only needs to assume that every spike of a neuron that participates in the population coding for one of the RVs *y^k^* sets the value of *y^k^* for a time period of length *τ* (= standard length of an EPSP) equal to the value encoded by this neuron. Then a suitably programmed network N of spiking neurons that results from recursive combinations of the basic module from Figure 2 can represent any distribution p^*^ through its spontaneous firing activity (provided that each module for a RV *y^k^* represents $p^{*}(y^{k}|y^{B(k)})$ as described above). If one decodes the current firing activity in the network N at any time *t* by setting each RV *y^k^* to that value that is indicated by the most recent firing of a neuron in the population code for *y^k^*, the resulting distribution of value assignments to the RVs *y* ^1^,  . . . , *y^K^* of p^*^ over time is exactly the one given by p^*^. In other words, p^*^ is the stationary distribution of the Markov chain that is defined by this network N of stochastically firing neurons. On the side, we would like to point out that this holds only after some initial “burn-in” phase, during which the distribution of network states becomes independent of the initial network state (Habenschuss et al., 2013a).

**Figure 5.**
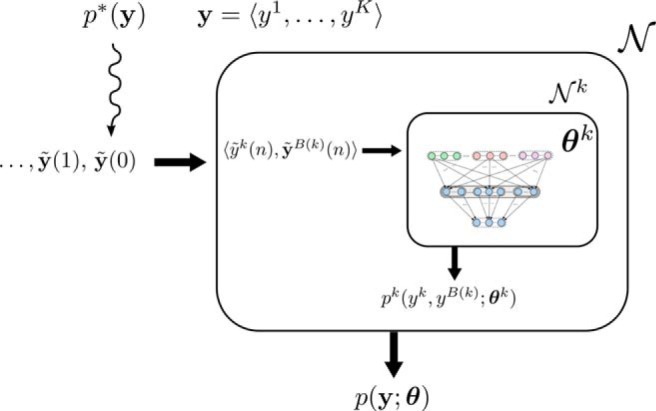
Schematic description of the learning approach. Sequence of examples $\tilde{y}(0),\tilde{y}(1),\;.\;.\;.$ drawn from the target distribution $p^{*}(y)$ are presented to the neural network N. The neural network is composed of learning modules $N^{k}$, one for each RV *y^k^*. $N^{k}$ learns from the components $〈{\tilde{y}}^{k}(n),{\tilde{y}}^{B(k)}(n)〉$ of examples $\tilde{y}(n)$ an approximation $p^{k}(y^{k},y^{B(k)};\theta^{k})$ of $p^{*}(y^{k},y^{B(k)})$ as indicated in Figures 2 and 3. The theory based on EM ensures that the total network N learns in this way an approximation p(y;  θ) of $p^{*}(y)$.

We now show (Fig. 5) that if one takes the previously analyzed learning capability of the basic network modules $N^{k}$ into account, the composed spiking network N learns from examples $\tilde{y}(n)$ of value assignments to $〈y^{1},\;.\;.\;.,y^{K}〉$ drawn from p^*^ values θ for its weights and biases that provide an approximation p(y;  θ) of *p* ^*^(**y**). This approximation p(y; θ) is represented by the network in the form of its stationary distribution of network states that result from its spontaneous firing activity. In order to achieve that, one just needs to allow each learning module $N^{k}$ to learn in parallel from those components of the example $\tilde{y}(n)$ that concern the random variables that it represents in the previously described manner. More precisely, each module $N^{k}$ receives the components $〈{\tilde{y}}^{k}(n),{\tilde{y}}^{B(k)}(n)〉$ of each example $\tilde{y}(n)$ that is presented to the network (for *n* = 1, 2,  . . . ). The network N learns an approximation of p^*^ from examples $\tilde{y}(n)$ without any additional computational overhead or teaching signals. Each subnetwork $N^{k}$ learns through STDP and intrinsic plasticity an internal model $p^{k}(y^{k},y^{B(k)};\;\theta^{k})$ of the marginal distribution $p^{*}(y^{k},y^{B(k)})$, as described in the preceding section.

One can rigorously prove that the sum over *k* of Kullback–Leibler divergences between the marginal distributions $p^{*}(y^{k},y^{B(k)})$ and the learnt internal models $p^{k}(y^{k},y^{B(k)};\;\theta^{k})$ converges through these adaptive processes to a local minimum (see Materials and Methods, Theorem 2). In this sense the spiking network N learns an approximation p(y;  θ) of the distribution $p^{*}(y)$.

As long as the RVs **y** are not split into inputs and outputs, this learning process is a typical example of unsupervised learning (see the definition in standard textbooks, such as Bishop (2006); Murphy, (2012); Haykin (2009)). Characteristic for unsupervised learning is that learning progress is measured in terms of the deviation between the learnt distribution p(y; θ) (= learnt internal model) and the distribution $p^{*}(y)$ from which the examples are generated. One also refers to this type of learning as density estimation.

Unsupervised learning has previously already been studied in a large number of artificial neural networks models: from Boltzmann machines (Ackley et al., 1985), neural belief networks (Neal, 1992), up to deep learning networks (Salakhutdinov and Hinton, 2012; Bengio et al., 2015). One major motivation of this work has been to discover learning principles of the brain, based on the argument that supervision for learning is rare in the brain. Our learning model provides a complementary approach, with the main difference being that it is based on networks of spiking neurons, rather than artificial neural networks, and that it uses STDP as primary plasticity mechanism. A major difference between the learning process in Boltzmann machines and our model is that our model does not require separate sleep phases. Its learning process is more similar to parameter learning in Bayesian networks (Koller and Friedman, 2009, Chapter 17). There the learning process also amounts to learning for each RV separately and in parallel from examples the conditional probability table for each RV, conditioned on the values of its parents. Such learning of a conditional probability table is analogous to the learning in a stochastic association module, except that such association module considers all RVs in the Markov blanket of a given RV, rather than just its parents. However, our learning approach is more general than parameter learning in Bayesian networks insofar, as it also encompasses aspects of structure learning (Koller and Friedman, 2009, Chapter 18), see section below, “Small numbers of hidden neurons in the learning modules often suffice.”

**Figure 6.**
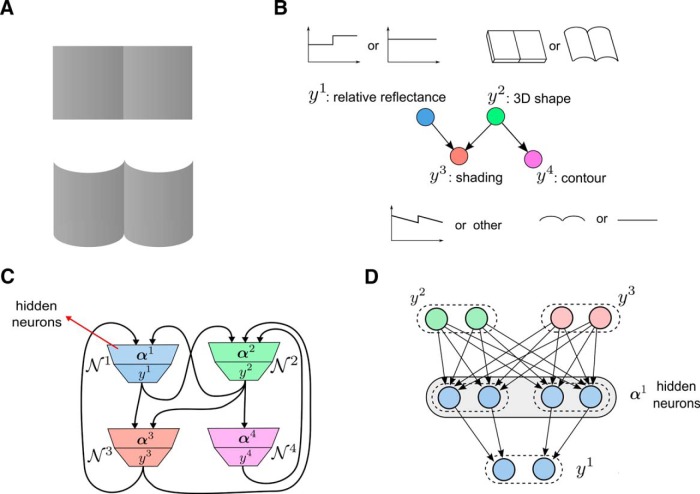
Description of the perceptual explaining away example. ***A***, The two visual stimuli used in the experiment from Knill and Kersten (1991). Both surfaces, top and bottom, have identical shading profiles in the horizontal direction. Nevertheless, subjects perceive that the reflectances of the two halves of the bottom panel are the same, whereas they perceive the left half of the top panel as being darker than the right half. The different contours of the two panels suggest different 3D shapes (flat vs cylindrical), which influences subjects’ perception of the reflectance of the two halves of each surface. ***B***, The “explaining away” Bayesian network proposed by Kersten and Yuille (2003) that models the effect from ***A***. It consists of four RVs *y* ^1^, *y* ^2^, *y* ^3^, and *y* ^4^. The relative reflectance *y* ^1^ of the surfaces can have two values: *y* ^1^ = 2 for different and *y* ^1^ = 1 for the same reflectance of the two parts of the surface. The 3D shape of the surfaces (*y* ^2^) is either cylindrical (*y* ^2^ = 2) or rectangular (*y* ^2^ = 1). The relative reflectance and the 3D shape are direct causes of the shading or the luminance change of the surfaces (*y* ^3^), which can have the profile like in the bottom part of ***B*** (*y* ^3^ = 2) or a different one (*y* ^3^ = 1). The 3D shape of an object causes different 2D contours (*y* ^4^), which can be either straight (*y* ^4^ = 1) or curved (*y* ^4^ = 2). The observed variables are the contour (*y* ^4^) and the shading (*y* ^3^) of the surfaces. Subjects infer the value of the relative reflectance (*y* ^1^) and the 3D shape (*y* ^2^) based on these observed cues. ***C***, The structure of the neural network N that corresponds to the Bayesian network in ***B***. For each RV *y^k^* in the Bayesian network there is a learning module $N^{k}$ composed of a population of neurons that outputs *y^k^* in population coding, and a population of hidden neurons $\alpha^{k}$. The learning modules are interconnected according to the Markov blankets of the RVs in the Bayesian network as indicated in Figure 4. For example, the RVs in the Markov blanket of *y* ^1^ are *y* ^2^ and *y* ^3^, and therefore the learning module $N^{1}$ receives connections from $N^{2}$ and $N^{3}$. ***D***, Structure of the learning module $N^{1}$ for the RV *y* ^1^ in the neural network in ***C***.

**Figure 7.**
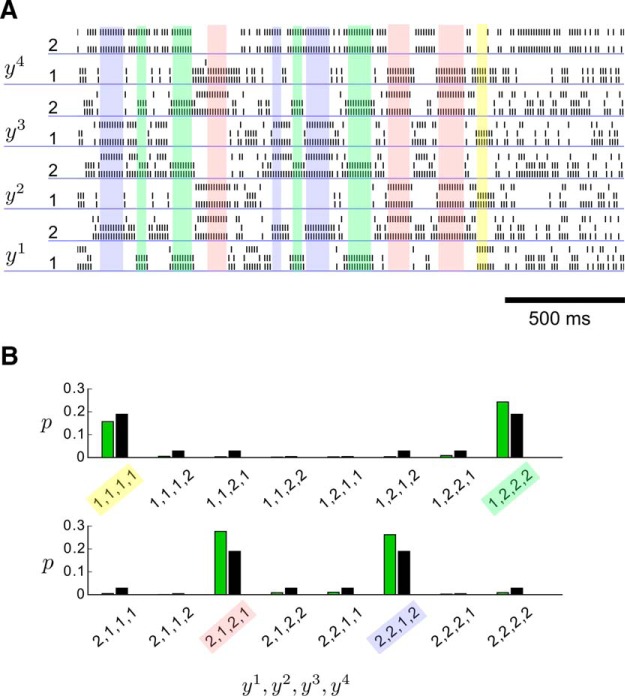
Analysis of the stationary distribution of network states of the network from Figure 6C after learning. ***A***, Random sample of spontaneous activity of the network (without any external input). The network switches stochastically between different network states (some of which are labeled by colors), in a manner that is qualitatively similar to experimental data from networks of neurons in the cortex (Abeles et al., 1995; Jones et al., 2007). The spike trains of neurons that encode a problem variable *y^k^* through population coding are underlined by blue lines. Spike trains immediately above are from the hidden neurons $\alpha^{k}$ that drive this neuron to fire (as in Fig. 2). ***B***, Frequency of network states that encode particular assignments to the problem variables *y^k^* (shown on the *x*-axis) resulting from the learned stationary distribution (spontaneous activity) p(y;  θ) of the network are shown as green bars. The coloring of the network states labels is the same as for corresponding network states in ***A***. Black bars indicate the probabilities of the same value assignments under the distribution p^*^ that had produced the examples for learning.

**Figure 8.**
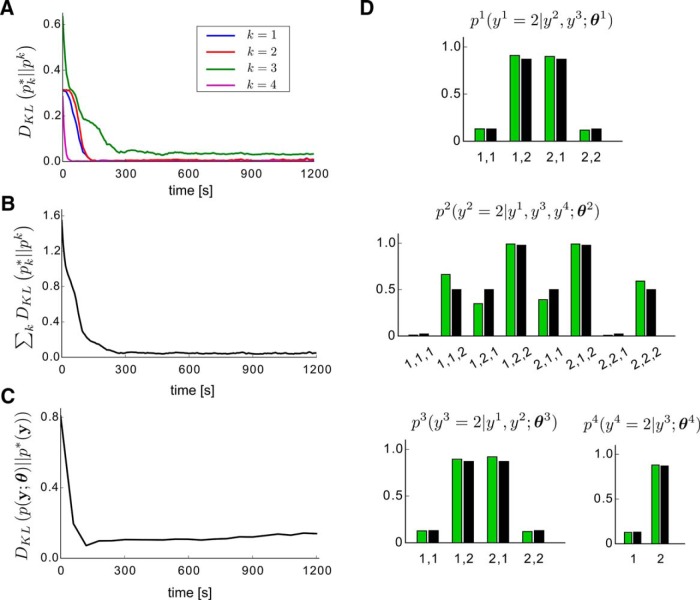
Performance of the recursive combination of learning modules from Figure 6*C* in learning the perceptual inference task of Knill and Kersten (1991) from examples. ***A***, Evolution of the KL divergence between the target distribution $p^{*}(y^{k},y^{B(k)})$ of the examples (denoted in the plot as $p_{k}^{*}$) and the distribution of the internal model $p^{k}(y^{k},y^{B(k)};\theta^{k})$ during learning, for each of the learning modules $N^{k}$ in the network (k=1,2,3,4). ***B***, Evolution of the sum of the KL divergences shown in ***A***. ***C***, Evolution of the KL divergence between the internal model distribution p(y;  θ) represented by the whole network and the target distribution $p^{*}(y)$ of the examples during learning. ***D***, The plots show the target conditional distributions $p^{*}(y^{k}|y^{B(k)})$ (black bars), and the learned conditional distributions $p^{k}(y^{k}|y^{B(k)};\theta^{k})$ (green bars), for each problem variable *y^k^*. The bit string labels on the *x*-axis denote the assignment of values to the problem variables on which each distribution is conditioned.

**Figure 9.**
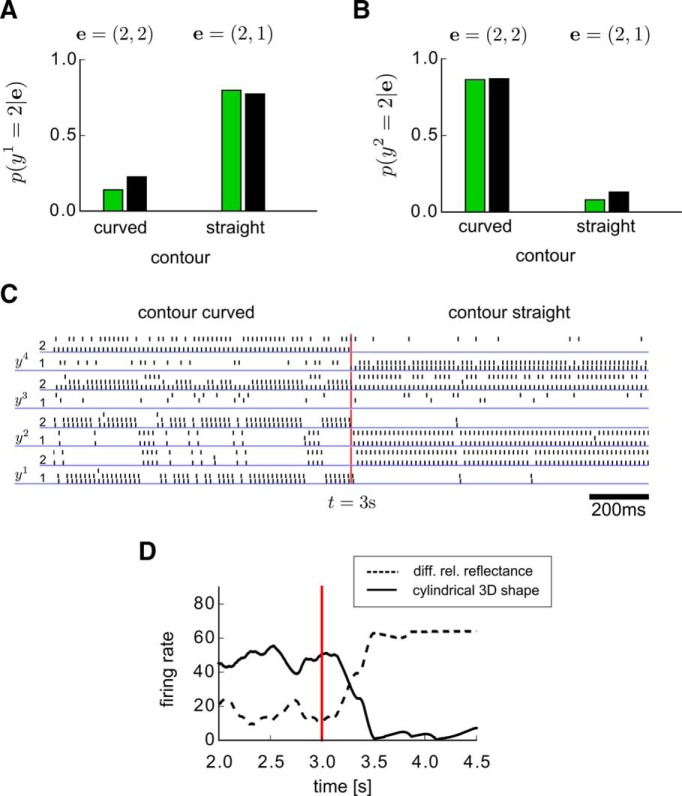
Demonstration that the network N from Figure 6*C* has learnt explaining away. ***A***, ***B***, Results of probabilistic inference (estimate of posterior marginal probabilities) by the network through observation of the firing rates of neurons that encode *y* ^1^ and *y* ^2^ (whereas other neurons are “clamped” to encode the evidence **e**) are shown as green bars. The results are from a single run with duration 400 ms per evidence value. Black bars indicate corresponding values for p^*^ (“ground truth”). The network estimates the probabilities $p(y^{1}=2|e;\;\theta )$ (indicating different relative reflectance according to Figure 6*B*) and $p(y^{2}=2|e;\;\theta )$ (indicating a cylindrical 3D shape) for two different evidence values **e** = (*y* ^3^, *y* ^4^) through sampling from its stationary distribution of network states. In the first inference we assume **e** = (2, 2) (indicating an observation with discontinuous shading of the object and curved contour), and in the second **e** = (2, 1) (observation of discontinuous shading and straight contour). ***C***, The spiking activity of the network N during online inference of hidden causes, where the evidence **e** was switched after *t* = 3s (red vertical line) from **e** = (2, 2) to **e** = (2, 1). Spike trains of neurons from population codes for problem variables *y^k^* are underlined by blue lines (as in Figure 7*A*). ***D***, Firing rates (estimated with a sliding alpha kernel $K(t)=\frac{t}{\tau }\;\exp\;(\frac{t}{\tau })$, *t* = 0.1 s) of the neurons encoding *y* ^1^ = 2 (different relative reflectance, dashed line) and *y* ^2^ = 2 (cylindrical 3D shape) during the inference simulation run shown in ***C***. The “explaining away” effect is clearly visible from the complementary evolution of the firing rates of the two neurons that represent the two potential hidden causes “cylindrical 3D shape” and “different relative reflectance.”

Like other generative models for unsupervised learning, our model also aims at extracting underlying structure in the training examples (Hinton et al., 1995), so that it can even generate fake examples that share the discovered underlying structure (Fig. 7). On the level of higher cortical areas such unsupervised learning could detect relationships between different types of features (Figs. 6–9), between object representations in different sensory modalities, or how an action modifies the environment.

In contrast to the previously mentioned paradigms for unsupervised learning in neural networks, and similar to parameter learning in Bayesian networks, the architecture that we are proposing has a clear modular structure (Fig. 5). It consists of stereotypical network motifs $N^{k}$ that each try to determine for one of the RVs *y^k^* to what extent values for *y^k^* can be predicted from the values of other RVs (more precisely: the RVs in its Markov blanket $y^{B(k)}$). As soon as this prediction becomes better than chance, the learning module $N^{k}$ has discovered some underlying structure in the examples $\tilde{y}$. One curious feature of this local prediction learning is that the learning process looks from the perspective of the learning module $N^{k}$ like supervised learning, because ${\tilde{y}}^{k}$ is the prediction target for input ${\tilde{y}}^{B(k)}$ to this module, and both ${\tilde{y}}^{k}$ and ${\tilde{y}}^{B(k)}$ are part of a training example $\tilde{y}$. This holds in spite of the fact that the whole examples $\tilde{y}$ are in general presented to the network without any supervision, ie, without any associated target output. This feature of the learning process is shared with parameter learning in Bayesian networks, where the learning of a conditional probability table for a RV *y* may look locally like supervised learning, because both the values of its parent nodes and the value of *y* are extracted from each training example.

Boltzmann machines and probabilistic graphical models such as Bayesian networks that are usually trained through unsupervised learning can, however, also be used for supervised learning (Hinton et al., 2006). In that case, the RVs **y** are split into two subsets **y***_I_* and **y***_O_*, ie, the target output ${\tilde{y}}_{O}$ is combined with the vector ${\tilde{y}}_{I}$ to form the examples $\tilde{y}=〈y_{I},y_{O}〉$ used for training. The goal is to learn a mapping from **y***_I_* to **y***_O_*, ie, to learn the distribution $p^{*}(y_{O}|y_{I})$. We consider here the general case where the mapping from inputs to target values is stochastic, either due to present noise in the target values or their inherent stochastic relation to the inputs. Typical classification problems where the mapping is assumed to be a deterministic function represent a special case of the stochastic formulation. For example, for supervised learning of image categorization in Boltzmann machines one simply adds the target category like an additional feature to the feature vector of an image. The learning process remains exactly the same (ie, unsupervised learning from examples $\tilde{y}$), and for learning one does not have to tell the network which variables are inputs and which are outputs. The only difference is that after learning only the components **y***_I_* of new test examples are provided and the network has to produce a guess for values of the components **y***_O_*. The same principle applies to the neural network in our approach: If **y** is partitioned into **y***_I_* and **y***_O_* it can learn the input–output mapping by learning an internal model of the full joint distribution $p^{*}(y_{I},y_{O})$ of the examples. After the learning process has finished, the network can estimate the probabilities of the target values $p^{*}(y_{O}|y_{I})$ given a particular input **y***_I_*. However, note that, as it learns the joint distribution, the network can additionally also answer any other probabilistic inference queries based on the probability distribution p^*^ of the examples (see section “Flexible retrieval of learnt statistical information through probabilistic inference”).

Classical learning of associative memory (like in a Hopfield network) also appears as a special case of the type of network learning that we are investigating: If $p^{*}(y)$ is nonzero only for some set $\tilde{y}(0),\;.\;.\;.,\tilde{y}(M-1)$ of *M* memory items. For these memory items the network learns then input completion. Such input completion can be accomplished through the learnt internal model p(y;  θ) in our framework: If one clamps some of the neurons to the values of some given incomplete pattern y^I, and lets the other neurons fire according to the stochastic dynamics of this internal model. But note that the learning task of our model is more demanding than classical learning of associative memory, because it also has to learn the probability distribution (frequency) of the patterns $\tilde{y}(n)$.

The underlying learning theory for our model (based on Expectation Maximization) does not guarantee that the internal model p(y; θ) converges to the target distribution $p^{*}(y)$. Rather, as in all known cases of nontrivial unsupervised learning and self-organization it can only guarantee that a local optimum is achieved (which cannot get worse if learning continues). However, even this weak form of a theoretical guarantee is actually quite rare in the literature on neural network learning via STDP. Most known successful methods for unsupervised learning or self-organization in machine learning are supported theoretically in the same weak manner via Expectation Maximization. However many of these methods work very well in practice. The learning speed and quality depend for the learning framework that we have introduced on the nature of the target distribution p^*^. We are demonstrating in Example 2 that this learning scheme works well for an example of p^*^ where it is well known that humans are able to learn a good approximation of probabilistic inference for p^*^.

### Flexible retrieval of learnt statistical information through probabilistic inference

After learning, the network N from Figure 5 has an approximation p(y;  θ) to $p^{*}(y)$ as its stationary distribution. This holds under the convention that the firing of a neuron that represents a specific value *l* of a variable *y^k^* sets this variable to value *l* for a duration *τ* equal to the duration of a generic EPSP. The learned distribution is manifested in the spontaneous activity of the network, ie, when no neurons in the network are clamped. Information can be extracted from this learnt internal model p(y;  θ) through probabilistic inference via neural sampling. This type of information retrieval goes far beyond input completion, which is the only form of information retrieval in classical neural network models for memory. In particular, after inserting evidence into N by exciting or inhibiting some of the neurons that represent a subset **y***_e_* of the variables, the spiking activity of the rest of the network generates according to Pecevski et al. (2011) samples from the conditional posterior distribution $p(y_{s}|y_{e};\;\theta )$, where **y***_s_* is the subset of variables that are not in **y***_e_*. Furthermore, the posterior marginal probabilities $p^{*}(y^{k}|y_{e})$ of the variables *y^k^* in **y***_s_* can be read out from the resulting firing rates of the neurons that represent these variables. Thus, information gathered from the examples $\tilde{y}(n)$ that had been presented to the network N (Fig. 5) can be extracted from this network in very flexible ways through probabilistic inference. This will be demonstrated for an example in Figures 6–9. In particular, the network can produce estimates of posterior marginal probabilities of the type indicated through examples in the Significance Statement. Note that these marginal probabilities, that are represented by the firing rates of corresponding neurons in N, integrate automatically information from many modes of the learnt approximation p(y;  θ) to p^*^. Hence, if these modes represent individual memory items of a memory model, the network N can combine information from many different memory items (episodes), also in ways that could not be anticipated during learning.

### Small numbers of hidden neurons in the learning modules often suffice

The structure of the network N in Figure 5 is very similar to the structure of a constructed network of spiking neurons that directly mimics a representation of p^*^ by a Bayesian network according to Pecevski et al. (2011). However, there the number of hidden neurons $\alpha^{k}$ for a random variable *y^k^* was required to be exponentially large in the number of variables in the Markov blanket of *y^k^*. In contrast, in the learning approach of this article, one can employ in principle any number, also a very small number, of hidden neurons in $\alpha^{k}$. The described learning approach will approximate the marginal distribution of p^*^ over *y^k^* and the Markov blanket of *y^k^* with a mixture distribution whose number of modes is determined by the chosen number of hidden neurons in $\alpha^{k}$. For example, in Example 1 we had chosen just two hidden neurons in α for each of the two possible values of *z*, instead of two times four that were used in the construction of Pecevski et al. (2011) for representing all four possible assignments of values to the inputs **x** of a module. But as the comparison of Figure 3, B and C (and of D and E) shows, the network with the smaller number of hidden neurons α works in this case about as well as the larger network. This effect is predicted from general results in learning theory: a learning network with fewer parameters sacrifices representation power, but gains generalization capability. Furthermore, naturally occurring distributions can often be approximated quite well by mixture distributions with a relatively small number of components (modes). This suggests that real world distributions p^*^ of examples can often be learnt by relatively small networks N. An ideal scenario from a biological perspective would be one where a population of hidden neurons in N can become larger if the size provides insufficient resolution or prediction capability for the examples that it receives.

Note that with this approach a spiking network N can in principle learn an approximation of a given distribution p^*^ even without prior information on the dependency structure among RVs of p^*^. One can set up the network N so that each module $N^{k}$ extracts the probabilistic association between RV *y^k^* and *all* other RVs $y^{\backslash k}$ (ie, replacing **y***^B^* ^(^*^k^* ^)^ by all RVs other than *y^k^*). The size (ie, number of hidden neurons α; Fig. 2) of $N^{k}$ determines the quality of the resulting learnt approximation of $p^{*}(y^{k}|y^{\backslash k})$. Whereas a good approximation can only be theoretically guaranteed if the number of hidden neurons in $N^{k}$ is exponential in the number *K* of RVs of p^*^, acceptable results may emerge with drastically fewer hidden neurons, provided that their number is in the same range as the sum of the number of main modes of the distributions $p^{*}(y^{\backslash k}|y^{k}=l)$ for different values *l* (Fig. 3 provides an illustration).

### Example 2: autonomous learning of explaining away in perceptual inference

We demonstrate learning in recursive combinations of the basic learning module for a concrete example with four modules (Fig. 6*C*). We apply it to the task of learning a complex distribution p^*^ that represents a standard example for explaining away in visual perception. The famous experiment of (Knill and Kersten, 1991), depicted in Figure 6, had first demonstrated that nontrivial inference is involved in visual perception. A subsequent study (Kersten and Yuille, 2003) proposed that this perceptual effect can be understood as “explaining away” in probabilistic inference, and a Bayesian network with 4 RVs, $y^{1},\;.\;.\;.,y^{4}$ (Fig. 6*B*) was introduced to demonstrate this. The probability distribution of the Bayesian network is $p(y^{1},y^{2},y^{3},y^{4})=p(y^{1})p(y^{2})p(y^{3}|y^{1},y^{2})p(y^{4}|y^{2})$, and the inference task is to calculate the marginal posterior probability distributions $p(y^{1}|y^{3},y^{4})$ and $p(y^{2}|y^{3},y^{4})$.

In the experiment of Knill and Kersten (1991), the demonstrated different perception of the shading in the two surfaces in Figure 6*A* (see legend for more details) can be explained by two different competing causes, either by different relative reflectance of the two abutting surfaces (*y* ^1^), or by their cylindrical 3D shape (*y* ^2^). An observed curved contour of the surfaces is a cue that increases the probability of a cylindrical 3D shape. Because a cylindrical 3D shape alone is enough to explain the shading, it reduces the probability that the relative reflectance is different. Hence, one of the competing causes, the cylindrical 3D shape, “explains away” the other possible cause, the different reflectance of the surfaces. In the other case, when a flat contour is observed as an additional cue, this increases the probability of a rectangular 3D shape. As a rectangular 3D shape cannot explain the observed shading, the probability of the second possible cause for the shading, the different reflectance is increased. This type of explaining away in probabilistic inference can only occur for distributions p^*^ that have higher-order interactions between three or more RVs, like between the two competing causes and the observed shading in this example.

We show that the underlying distribution p^*^ can be learnt (approximately) from examples for this visual perception task, and that the network N which learns this approximation learns simultaneously to deal with the explaining away effect as an emergent phenomenon.

The structure of the neural network N suitable for learning this target probability distribution p^*^ is given in Figure 6*C*. It consists of four interconnected learning modules, where the connections between the learning modules reflect the dependencies between the RVs in the Bayesian network in Figure 6*B*. Additionally, the structure of one of the learning modules, the learning module $N^{1}$ for the RV *y* ^1^, is given in Figure 6*D* in detail. Each subgroup $\alpha^{1l}$ of hidden neurons has two neurons. This number of hidden neurons is smaller than the number of hidden neurons in the exact neural implementations of this Bayesian network by Pecevski et al. (2011), (their Implementation 2), equal to the total number of assignments of values to the RVs in the Markov blanket, which in this case is four. But we show that the smaller neural network can nevertheless learn the distributions $p^{*}(y^{2},y^{3}|y^{1}=l)$, because these distributions do not have more than two modes. In fact, we use here just two hidden neurons in the subgroups $\alpha^{kl}$ of all learning modules, also for the learning module $N^{3}$, where the total number of assignments of values to the RVs in the Markov blanket is 8. As we will see in the results, two hidden neurons in the learning module $N^{3}$ are enough to learn a good approximation of $p^{*}(y^{1},y^{2},y^{4}|y^{3}=l)$.

We performed computer simulations of learning with this network, where examples drawn from the target probability distribution p^*^ were presented to the network successively during learning. The distribution p^*^ was defined according to Table 4 in Materials and Methods in order to capture the visual perception scenario of Figure 6 in a qualitative manner. The learning phase took 1200 s of simulated biological time, and each example was presented for a time period of 100 ms. The weights and biases of the neurons were randomly initialized before learning.

We first analyzed the stationary distribution of network states in the network N from Figure 6*C* after learning. Figure 7*A* shows that the network switches spontaneously between different network states, and occasionally remains longer in one of the network states that have high probability under the stationary distribution (Fig. 6*B*). One can relate the firing activity of this network N to an approximation p(y;  θ) of the target distribution $p^{*}(y)$ by assuming in the usual manner (Berkes et al., 2011; Buesing et al., 2011) that the firing of a neuron in the population code for variable *y^k^* sets the value of this variable for a time period of length *τ* to the value encoded by this neuron. The distribution over 4 binary random variables $y^{1},\;.\;.\;.,y^{4}$ obtained in this way from the spontaneous firing of the network N is shown in Figure 7*B* and compared with the target distribution p^*^.

In Figure 8*A* we examined how the learning in the modules progresses in time. In each of the four learning modules in the network (one for each problem variable *y^k^*; Figs. 5, 6*C*) the Kullback–Leibler (KL) divergence between the marginal target distribution $p^{*}(y^{k},y^{B(k)})$ that it learns to approximate and its internal model $p^{k}(y^{k},y^{B(k)};\;\theta )$ decreases and stabilizes to a local minimum after about 300 s. The same is true for the sum of all KL divergences of the modules (Fig. 8*B*). As a result, the difference between the model distribution p(y;  θ) of the network and the full target distribution $p^{*}(y)$ also decreases, as shown in Figure 8*C*. This is because after learning a good internal model of the marginal target distributions $p^{*}(y^{k},y^{B(k)})$, the firing probability of the output neurons of the learning modules approximate well the conditionals $p^{*}(y^{k}|y^{B(k)})$ of p^*^, as shown in Figure 8*D*, which according to the theory leads to a good approximation of p^*^ by the network.

The PSPs and STDP curves in the simulated network have a more biological smoother alpha shape, which differs from the rectangular shape used in the theory. This can introduce minor deviations of the learning convergence from the theoretically optimal one, as for example the slight increase of the KL divergence in Figure 8*C* in the second half of the learning process.

This network N can extract the information that it has acquired from the examples in a very flexible manner through probabilistic inference. For example, if evidence **e** is entered for some of the problem variables $y^{1},\;.\;.\;.,y^{4}$ (by inducing corresponding neurons in their population codes to fire at high rates) the conditional marginal probabilities of other variables *y^i^* can be read off from the firing rates of neurons that represent *y^i^* through population coding. In particular, we demonstrate in Figure 9 that the network N has acquired autonomously from examples the capability to carry out nontrivial probabilistic inference that involves explaining away (ie, higher order dependencies among random variables).

Finally, we would like to point out that our approach is not restricted to Bayesian networks; it can be applied to any type of graphical models, eg, also to Markov random fields and factor graphs. The network connectivity is determined by the Markov blankets of the random variables, which can easily be read out from the graph structure of any graphical model.

## Materials and Methods

We first present a rigorous learning theory that supports the learning results that are presented. After that, we provide details to the computer simulations.

### Theoretical properties of the basic learning module (stochastic association module) and its plasticity

In this section, we give additional details about the structure of the module, the firing of the output neurons and the learning procedure. In particular, the subsequent subsection titled “Implicitly represented generative model” defines the probabilistic model p(x,z;  θ) represented in the module. After that, in the two subsections “Firing probability of the output neurons resulting from the internal generative model” and “Deriving the probability distribution of the firing of the neurons α” the firing of the output and the alpha neurons in the module is expressed through the represented probabilistic model. The last subsection “The plasticity rules minimize the KL divergence through Expectation Maximization” continues with the explanation how the plasticity rules implement learning of the stochastic associations between the variables **x** and *z* through EM.

The neurons α in the learning module are interconnected through inhibitory interneurons (data not shown in Fig. 2) that enforce strong lateral inhibition between them. The role of the lateral inhibition is to ensure that if one of the α neurons fires at time *t*, no other neuron in α will fire within the interval (*t*, *t* + *τ*). Each neuron in population α receives input synaptic connections from all input neurons χ such that the firing of the population $\chi^{i}$ encodes the value of the input variable *x^i^* (Fig. 2). For each non-zero value of the input variable *x^i^* = *m* there is a dedicated neuron $\chi^{im}$, and if $\chi^{im}$ fired in the time period (*t* – *τ*, *t*], the value of *x^i^* at time *t* is *x^i^* = *m*. The firing in the population $\chi^{i}$ is such that no two neurons fire in the time interval (*t* – *τ*, *t*], ie, after a spike there is no spiking period of duration *τ*. If there is no spike in the interval (*t* – *τ*, *t*], then the value of *x^i^* is 0.

All neurons in the population $\alpha^{l}$ connect to their corresponding output neuron *ζ^l^*. These synaptic connections are not subject to synaptic plasticity and have a fixed strong efficacy. The strong weights of these synapses achieve that whenever any of the neurons in $\alpha^{l}$ fires, it drives the output neuron *ζ^l^* to fire. The output neurons fire only if they receive input spikes, otherwise they remain silent. Thus, the spikes that the neuron *ζ^l^* emits can be seen as a union of all spikes of the hidden neurons in the group $\alpha^{l}$.

One can give an analytical description for the firing probability density of the output neuron *ζ^l^* according to the model. To do that, first we need to introduce some notation. With $w_{im,j}^{l}$ we denote the efficacy of the synaptic connection from the input neuron *χ^im^* to the neuron $\alpha_{j}^{l}$ from the group $\alpha^{l}$. For the bias (intrinsic excitability) of the neuron $\alpha_{j}^{l}$, we write $b_{j}^{l}$. We also introduce, for simplicity, binary RVs *x^im^* corresponding to each neuron *χ^im^*. The binary RV *x^im^* assumes value 1 at time *t* if the current value of the RV *x^i^* is equal to *m*, and 0 otherwise. The current value of *x^im^* at time *t* can be determined just from the spikes of the neuron *χ^im^*, ie, it is 1 if *χ^im^* fired within the time interval [*t*, *t* – *τ*], and 0 otherwise. With this notation, we can describe the firing probability density of *ζ^l^* at time *t* by:

(4) $$\rho^{l}(t)=\overset{J^{l}}{\underset{j=1}{\sum }}\frac{1}{\tau }\exp\;(u_{j}^{l}(t))=\overset{J^{l}}{\underset{j=1}{\sum }}\frac{1}{\tau }\exp\;(b_{j}^{l}+\overset{I}{\underset{i=1}{\sum }}\overset{M(x^{i})}{\underset{m=1}{\sum }}w_{im,j}^{l}\;x^{im}(t)),$$

where $u_{j}^{l}$ is the membrane potential of neuron $\alpha_{j}^{l}$, *J^l^* is the number of neurons in the subpopulation $\alpha^{l}$, and *M*(*x^i^*) denotes the maximum integer value that *x^i^* can assume. The firing probability density of the output neuron *ζ^l^* is equal to the sum of the firing probability densities of all neurons in the subpopulation $\alpha^{l}$, which follows from the specific connectivity structure in the learning module. The sums with the indices *i* and *m* iterate over all input neurons *χ^im^*.

During learning, we assume that examples $〈\tilde{z}(0),\tilde{x}(0)〉,〈\tilde{z}(1),\tilde{x}(1)〉,\;. . .,\;〈\tilde{z}(n),\tilde{x}(n)〉,. . .$ drawn from the joint probability distribution $p^{*}(z,x)$ are presented to the WTA circuit in succession, one by one. In an example the variable *z* has an integer value from the set {1, . . . ,M(z)} (*M*(*z*) denotes the maximum integer value that *z* can assume), and each of the variables *x^i^* assumes a value from the set $\left\{1,. . .,M(x^{i})\right\}$. Each example $〈\tilde{z}(n),\tilde{x}(n)〉$ is presented for several tens or hundreds of milliseconds. During this time period, the input neurons fire according to the values of the input variables $\tilde{x}(n)$. In particular, if ${\tilde{x}}^{i}(n)=m$, then the input neuron *χ^im^* fires with a high firing rate, whereas all other input neurons in the population $\chi^{i}$ are silent. The value $\tilde{z}(n)$ in the example is given to the network via inhibitory currents in a subset of the neurons α. More precisely, if in the current example $\tilde{z}(n)=l$, then all neurons in α that do not belong to $\alpha^{l}$ are inhibited with a strong negative external current which prevents them from firing. At the same time the neurons in the group $\alpha^{l}$ do not receive any external inhibitory currents and are free to fire according to their firing probabilities determined by their inputs. At the beginning of the learning process, it is assumed that the biases of the α neurons have large values so that they fire with high firing rates regardless of what the presented input $\tilde{x}(n)$ is.

As stated above, in the presented examples during learning the variables *z* and *x^i^* (*i* = 1, . . . , *I*) assume values from the sets {1, . . . ,M(z)} and $\left\{1,. . .,M(x^{i})\right\}$, respectively. These values could represent states of the external environment, internal beliefs or any other behavior related variable value. However, in the population coding of values of the variables *z* and *x^i^* (*i* = 1, . . . , *I*) by their corresponding neuron populations in the learning module, the variables can have an additional value of zero. A neuron population that encodes a variable through population coding assigns value zero to this variable if none of the neurons fires for a period longer than *τ*. There is not a dedicated neuron whose firing signals a zero value, as is the case for the rest of the non-zero values of the variable. The assumed spike based encoding with a zero value is the same as in the neural sampling theory (Buesing et al., 2011; Pecevski et al., 2011). It defines a value for the variable at each moment in the continuous time dynamics of the neural network model.

The zero values in the population coding of the learning module do not represent states of the external environment. The reason for this is that the learning module does not learn the probabilities p(x,z; θ) in its internal model where some of the variables *z* and *x^i^* (*i* = 1, . . . , *I*) have zero value. These probabilities have very fixed small (close to zero) values (see section “Firing probability of the output neurons resulting from the internal generative model”). Therefore, it is assumed that the examples contain only the encoded variable values whose stochastic relations can be learnt by the learning module.

#### Implicitly represented generative model

The learning theory of the module is based on its internally represented generative model p(z,x; θ), as it is discussed in Results, section “A network module for learning stochastic associations” (see also section “The plasticity rules minimize the KL divergence through Expectation Maximization”). In order to define the implicitly represented generative model, we first define an additional compound RV a in form of a vector of binary RVs, that we will relate to the stochastic activity of the neurons in the population α. More precisely, the RV a is defined as a population code, represented as a set of binary RVs $a_{j}^{l}$, one for each neuron $\alpha_{j}^{l}$. The generated values of $a_{j}^{l}$ over time during the activity of the WTA circuit are defined by the spiking of $\alpha_{j}^{l}$ in the same way as the spiking of the input neurons χ define the values of **x**. The value of $a_{0}^{0}$, which does not have a corresponding neuron in the population α, is defined as $a_{0}^{0}=1-\sum_{l,j}a_{j}^{l}$. Note that as there can be no two neurons in α spiking within the time window (*t* – *τ*, *t*) because of the lateral inhibition, it follows that the vector RV a has a restricted domain of allowed values, consisting only of values where exactly one $a_{j}^{l}$ is equal to 1, and the others are 0. The value of a where only $a_{j}^{l}=1$ and the rest are 0 will be denoted by a=(l,j).

Having defined a, we can now write the parametrized form of the generative model as follows:

(5) p(a,z,x; θ)==1A(θ) p(z|a) exp (∑i,m∑l, jw^im,jl xim ajl+∑l, jb^jl ajl) ,

where A(θ) is the normalization constant. In the sum over the indices *i* and *m*, the index *i* iterates through all input variables from *x* ^1^ to *x^I^*. For each value of the index *i*, the index *m* iterates from 0 to *M*(*x^i^*), ie, through the set of possible values of the RV *x^i^*. In the two sums with the indices *l* and *j*, *l* iterates through all possible values of *z* from 0 to *L*, and *j* iterates from 1 to *J^l^* (note that *J* ^0^ = 1, as there is not a group of neurons in α for *l* = 0, and hence only 1 parameter). The parameter vector θ consists of all parameters ${\hat{w}}_{im,j}^{l}$ and ${\hat{b}}_{j}^{l}$ which are encoded in the synaptic weights and biases of the alpha neurons. After learning, the marginal distribution p(z,x; θ) of Equation 5 becomes an internal model of the distribution $p^{*}(x,z)$ of the presented examples. The probability p(z|a) in Equation 5 is not parametrized and specifies the deterministic relations between a and *z*. It is defined as follows: $p(z=l_{1}|\text{a}=(l_{2},j))=1$ if *l*_1_ = *l*_2_, and = 0 otherwise, for all $l_{1}\in \{0,. . .,L\}$, and all (*l*_2_, *j*) that are valid values of a. These probability values express the fact that when the neuron $\alpha_{j}^{l}$ fires and sets the value of a to be (*l*, *j*), then this also uniquely determines *z*, ie, sets the value of *z* to *z* = *l*.

A sufficient condition for having a normalization constant A(θ)=1 are the following constraints on the parameters:

(6) ∑mexp (w^im,jl)=1    for all i∈{1, . . . ,I}, j∈{1, . . . ,Jl} and l∈{0, . . . ,L}, and ∑l∑jexp (b^jl)=1 .

As we will see later, one property of the plasticity rules is that they move the parameter vector toward the region of the parameter space where these normalization constraints are approximately satisfied, ie, they try to keep the internal probabilistic model normalized. If one assumes that the normalization constant satisfies A(θ)=1, then the generative model in Equation 5 obtains the form:

(7) $$p(\text{a},z,x;\theta )=p(\text{a};\theta )\;p(z|\text{a})\overset{I}{\underset{i=1}{\prod }}p(x^{i}|\text{a};\theta ).$$

By marginalizing a in the full generative model p(a,z,x; θ), we obtain the marginal probability distribution p(z,x; θ), which has the form of a mixture of multinomials. The marginal probabilistic model p(z,x; θ) models the probabilistic relations between the RV *z*, encoded by the firing of the output neurons, and the RVs **x**, which are encoded in the inputs. These relations are modeled through the vector of auxiliary RVs a, which are also called hidden RVs. In the mixture model the conditional probabilities p(z|a) and $p(x^{i}|\text{a};\theta )$ for *i* = 1, . . . , *I* are the likelihoods, and p(a; θ) is the prior.

It can be easily shown from Equation 5 that by assuming A(θ)=1, the likelihoods $p(x^{i}|\text{a};\theta )$ for *i* = 1, . . . , *I* are:

(8) $$p(x^{i}=m|\text{a}=(l,j);\theta )=\;=\exp\;({\hat{w}}_{im,j}^{l})\;\;\;\text{ for all}\;l\text{, }j\text{ and }m.$$

Similarly the priors are:

(9) $$p(\text{a}=(l,j);\theta )=\exp\;({\hat{b}}_{j}^{l})\;\text{ for all }l\text{, }j.$$

Hence, given that the normalization constraints in Equation 6 hold, the parameters ${\hat{w}}_{im,j}^{l}$ are equal to the log of the conditional probabilities from the likelihood ${\hat{w}}_{im,j}^{l}=\log\;p(x^{im}=1|a_{j}^{l}=1;\theta )$, whereas ${\hat{b}}_{j}^{l}$ represent the log probabilities of the prior ${\hat{b}}_{j}^{l}=\log\;p(a_{j}^{l}=1;\theta )$.

For convenience, Table 1 lists the mathematical notation that is used in the definition of the learning module.

**Table 1.** Mathematical symbols used in the definition of the learning module

**Symbols related to the RVs of the inputs and the outputs**	
**x**	Vector of all multinomial RVs $\left(x^{1},...,x^{I}\right)$ corresponding to the inputs
*x^i^*	*i*-th multinomial RV from the vector **x**
*z*	RV corresponding to the output neurons
*M*( . . . )	Operator that gives the maximum integer value of the RV given as an argument; for example *M*(*x^i^*) and *M*(*z*) denote the maximum values of *x^i^* and *z*, respectively.
$p^{*}(x,z)$	Target probability distribution learned by the learning module
**The output and input neurons in the learning module**	
$\chi^{i}$	Population of input neurons that together encode the value of the RV *x^i^* through population coding
*χ^im^*	Input neuron in $\chi^{i}$ whose firing signals the value *m* of the RV *x^i^*
*x^im^*	Binary RV that assumes value 1 if and only if *x^i^* = *m*; it corresponds to the coding property of the input neuron *χ^im^*.
ζ	Population of output neurons that encode the value of the RV *z*
*ζ^l^*	Output neuron in ζ whose firing signals the value *l* of the RV *z*
**The WTA populations of neurons in the learning module and their associated RVs**	
α	The whole WTA population of neurons that represent the auxiliary RVs a
$\alpha^{l}$	Subpopulation of neurons in α that connects to the output neuron *ζ^l^*
*J^l^*	Number of neurons in $\alpha^{l}$
$\alpha_{j}^{l}$	A neuron from the subpopulation $\alpha^{l}$
$a_{j}^{l}$	Binary RV which value corresponds to the coding property of the neuron $\alpha_{j}^{l}$
${\text{a}}^{l}$	Vector of all RVs $a_{j}^{l}$ (for all $j=1,...,J^{l}$) that corresponds to the subpopulation of neurons $\alpha^{l}$
a	Vector of the union of the RVs in the vectors ${\text{a}}^{l}$ for all l=0,...,L; corresponds to the WTA population α
**Synaptic weights and biases and their corresponding parameters in the generative model**	
$b_{j}^{l}$	Bias (intrinsic excitability) of the neuron $\alpha_{j}^{l}$
$w_{im,j}^{l}$	Synaptic weight of the synaptic connection that connects the input neuron *χ^im^* to the neuron $\alpha_{j}^{l}$
p(x,z,a;θ)	Probability distribution of the generative model implicitly represented in the module
${\hat{b}}_{j}^{l}$	Parameter in the generative model p(x,z;θ); every such parameter, except for *l* = 0, is represented in the learning module by the bias $b_{j}^{l}$ through the relation $b_{j}^{l}={\hat{b}}_{j}^{l}-b_{-}$.
${\hat{w}}_{im,j}^{l}$	Parameter in the mixture generative model p(x,z;θ); every such parameter, except the ones with *l* = 0 or *m* = 0, is represented in the network by the synaptic weight $w_{im,j}^{l}$ through the relation $w_{im,j}^{l}={\hat{w}}_{im,j}^{l}-w_{-}$.
θ	Vector of all parameters of the generative model of the module; it includes all ${\hat{b}}_{j}^{l}$ (for all *l* and *j*) and all ${\hat{w}}_{im,j}^{l}$ (for all *l*, *i*, *m* and *j*) as components.
**Indices used throughout all symbols**	
*l*	Index that iterates through the output neurons *ζ^l^*, and through their corresponding WTA subpopulations $\alpha^{l}$ as well as through the binary RVs *z^l^*
*j*	Index that enumerates the individual neurons in the subpopulation $\alpha^{l}$
*i*	Index that iterates through the RVs *x^i^*, and also through their corresponding populations of input neurons $\chi^{i}$
*m*	Index that enumerates the binary RVs *x^im^* that represent individual values of the input RV *x^i^*, and their corresponding input neurons *χ^im^* in the population $\chi^{i}$

#### Firing probability of the output neurons resulting from the internal generative model

Here we will express the firing probability density of the output neurons through the probabilistic model p(z,x; θ). This will show that by learning the internal model p(z,x; θ) of the target distribution $p^{*}(z,x)$, the output neurons actually learn to fire according to the conditional $p^{*}(z|x)$. Thus, as discussed in Results, section “A network module for learning stochastic associations,” the module exhibits the learned stochastic associations between the variables *z* and **x** through the firing of its output neurons given input **x**. Furthermore, this particular form of firing enables to recurrently interconnect multiple modules in larger networks to learn more complex distributions. Networks of interconnected learning modules are presented in Results, section “Recursive combinations of the basic learning module enable efficient learning of complex distributions from examples,” and further discussed in Materials and Methods, section “Theoretical properties of networks of recursively interconnected basic learning modules.”

As a prerequisite to the derivation, we first establish a relation between the parameters ${\hat{w}}_{im,j}^{l}$ and ${\hat{b}}_{j}^{l}$ of the probabilistic model p(a,z,x; θ), and the synaptic weights $w_{im,j}^{l}$ and biases $b_{j}^{l}$ of the neurons in α. For the synaptic weights $w_{im,j}^{l}$ we assume that they are equal to the corresponding parameters ${\hat{w}}_{im,j}^{l}$ shifted by a constant baseline value $w_{im,j}^{l}={\hat{w}}_{im,j}^{l}-w_{-}$. Similarly, the biases of the neurons α represent linearly translated values of the parameters ${\hat{b}}_{j}^{l}$, ie, $b_{j}^{l}={\hat{b}}_{j}^{l}-b_{-}$. The relation is defined through the following equations:

(10) $$\begin{array}{cc} & w_{im,j}^{l}={\hat{w}}_{im,j}^{l}-{\hat{w}}_{i0,j}^{l}-{\hat{w}}_{im,1}^{0}+{\hat{w}}_{i0,1}^{0},\text{ and }\\ & b_{j}^{l}={\hat{b}}_{j}^{l}-{\hat{b}}_{1}^{0}+\sum_{i=1}^{I}\left({\hat{w}}_{i0,j}^{l}-{\hat{w}}_{i0,1}^{0}\right). \end{array}$$

This entails that the modification of synaptic weights and biases via synaptic and intrinsic plasticity during learning results in adaptation of the parameters of the represented generative model. Not all parameters in the generative model are learned, however. We assume that the parameters:

(11) w^i0,jl for all i, all j and all l≠0,w^im,10 for all i, all m≠0,w^i0,10 for all i, and b^10 ,

that do not have a corresponding synaptic weight or a bias, have fixed values that are not subject to learning. In particular, ${\hat{w}}_{i0,j}^{l},\;{\hat{w}}_{i0,1}^{0}$ and ${\hat{b}}_{1}^{0}$ are assumed to have the following values:

(12) w^i0,jl= −V   for all l≠0, i and j, w^i0,10= −V   for all i, andb^10= −V ,

where *V* is a large positive constant. We assume that the constant *V* is large enough so that the probabilities p(z,x; θ) where at least one of the variables *z* and *x^i^* (for *i* = 1, . . . , *I*) has zero value are much smaller than the probabilities of value assignments where all variables have non-zero values (Eq. 5).

The fixed parameters are all subsumed in the baseline values *w*_–_ and *b*_–_:

(13) w− =  w^i0,jl+w^im,10−w^i0,10       for all i, j, l≠0 and m≠0, andb− =  b^10−∑i=1I(w^i0,jl−w^i0,10)     for all l and j  .

From this it follows that one can write the synaptic weights and the biases as:

(14) wim,jl = w^im,jl−w− ,bjl = b^jl−b− .

Hence, we have a one-to-one mapping between the synaptic weights and biases of the neurons α, and the parameters in the generative model that are subject to adaptation during learning. Note that from Equations 12 and 13, it follows that:

(15) w^im,10 = w−  for all i and m≠0, and b^10 = b− .

If we now substitute the synaptic weights and biases from Equation 10 in the expression for the firing probability density *ρ^l^*(*t*) of the output neuron *ζ^l^* from Equation 4, we obtain:

(16) ρl(t)==1τ·∑j=1Jlexp (b^jl+∑i=1Iw^i0,jl+∑i=1I∑m=1M(xi)(w^im,jl−w^i0,jl)xim(t))exp (b^10+∑i=1Iw^i0,10+∑i=1I∑m=1M(xi)(w^im,10−w^i0,10)xim(t)) .

In this expression, we can combine the two sums over the index *i* that iterates through 1, . . . , *I* (we do this both in the denominator and in the numerator), and by using the equality $1-\sum_{m=1}^{M(x^{i})}x^{im}(t)=x^{i0}(t)$ (where *x^i^* ^0^ is a binary RV, such that *x^i^* ^0^ = 1 if and only if *x^i^* = 0), we get the simplified form:

(17) $$\rho^{l}(t)=\frac{1}{\tau }\cdot \frac{\sum_{j=1}^{J^{l}}\exp\;\left({\hat{b}}_{j}^{l}+\sum_{i=1}^{I}\sum_{m=0}^{M(x^{i})}{\hat{w}}_{im,j}^{l}x^{im}\right)}{\exp\;\left({\hat{b}}_{1}^{0}+\sum_{i=1}^{I}\sum_{m=0}^{M(x^{i})}{\hat{w}}_{im,1}^{0}x^{im}\right)},$$

where we write simply *x^im^* instead of *x^im^*(*t*), as we assume it is implicitly understood that it is a function of time. It is clear that the denominator is equal to p(z=0,x; θ). In the numerator, we can rewrite the sum by iterating over values of the RV a as:

(18) ρl(t)=1τ·∑ap(z=l|a) exp (∑l′, j b^jl′ajl′+  +∑l′, j∑i=1I∑m=0M(xi)w^im,jl′ ximajl′)p(z=0,x; θ) A(θ)

where the sum indexed by a iterates over all possible values of the compound RV a having only one $a_{j}^{l}=1$ and others equal to 0, and in both sums inside the exp function indexed by l′ and *j*, the index l′ iterates over all possible values of *z* which are {0, . . . ,L}, and *j* iterates from 1 to $J^{l\prime }$. It is now easy to see that the numerator is equal to p(z=l,x; θ) A(θ), and finally we obtain:

(19) $$\rho^{l}(t)=\frac{1}{\tau }\cdot \frac{p(z=l|x(t);\theta )}{p(z=0|x(t);\theta )}\;.$$

Thus, the firing probability of the output neuron *l* is proportional to p(z=l|x; θ), and if the internal model p(x,z; θ) is close to $p^{*}(x,z)$, it will be proportional to $p^{*}(z=l|x)$ (as pointed out in Results, section “A network module for learning stochastic associations”). For how this result is used in the theory for networks of interconnected learning modules that learn to perform probabilistic inference in larger distributions see section “Theoretical properties of networks of recursively interconnected basic learning modules.”

#### Deriving the probability distribution of the firing of the neurons α

We show in this subsection that the firing of the neurons α in the WTA circuit during the presentation of an example $〈\overset{˜}{x}(n),\tilde{z}(n)〉$ generates samples from the distribution $p(\text{a}|\overset{˜}{x}(n),\tilde{z}(n);\theta )$. This result is used in section “The plasticity rules minimize the KL divergence through Expectation Maximization,” where the link between the plasticity rules, the activity of the neurons, and different calculations of the EM algorithm are discussed. In particular, it is explained there that, as the RVs a are the hidden variables in the probabilistic model, the samples generated by the activity of the α neurons together with the presented examples, form samples from the complete data distribution, which represents the expectation step of the EM algorithm. The result is further used in the section “Proof that the plasticity rules minimize the objective function U(θ) through Expectation Maximization” in that the proof is based on analyzing mean weight updates of the plasticity rules over the complete data distribution.

During the presentation of a single example, in the periods when it is not inhibited by the lateral inhibition due to a spike by another neuron in α, the firing probability density of each neuron $\alpha_{j}^{l}$ in α ideally remains constant over time. Let us denote the stationary distribution of its associated RV $a_{j}^{l}$ during the presentation of the example $〈\overset{˜}{x}(n),\tilde{z}(n)〉$ with $p_{\left(n\right)}(a_{j}^{l})$. We will also here use the assumption that the total firing rate of the $\alpha^{l}$ neurons is very high upon presentation of an example with $\tilde{z}(n)=l$ irrespective of what the input $\tilde{x}(n)$ is. That this assumption is true can be seen from the following. As we stated previously, we assume that the initial values of the biases of the α neurons are high. This implies that in the beginning of learning, for each presented example there will be active α neurons that fire in response to the example. In addition, as *b*_–_ has a large negative value (Eqs. 13, 15), it follows that the plasticity rule for the biases defined in Equations 2 and 3 will drive and stabilize the biases of the active α neurons toward very large values. Thus, when an example with $\tilde{z}(n)=l$ is presented, there will be always a subset of the neurons in α that have high firing rates. Consequently, during the presentation of the example almost all the time there will be a neuron in $\alpha^{l}$ active and $p_{\left(n\right)}(a_{1}^{0}=1)\approx 0$.

If we use the fact that $p_{\left(n\right)}(a_{1}^{0}=1)\approx 0$ for $p_{\left(n\right)}(a_{j}^{l})$ we can write:

(20) $$p_{\left(n\right)}(a_{j}^{l}=1)=\frac{\rho_{j}^{l}}{\overset{J^{l}}{\underset{j\prime =1}{\sum }}\rho_{j\prime }^{l}}\;\;\;\;\text{if}\;\;\;\overset{˜}{z}(n)=l\;,$$

and equal to 0 otherwise. Here $\rho_{j}^{l}$ denotes the firing probability density of $\alpha_{j}^{l}$, which is constant during the presentation of the example. If we substitute now the firing probability density in Equation 20 with:

(21) $$\rho_{j}^{l}=\frac{1}{\tau }\exp\;\left(b_{j}^{l}+\overset{I}{\underset{i=1}{\sum }}\overset{M(x^{i})}{\underset{m=1}{\sum }}w_{im,j}^{l}{\;\overset{˜}{x}}^{im}(n)\right)\;,$$

and additionally substitute the synaptic weights and biases with their related model parameters according to Equation 10, we get:

(22) p(n)(ajl=1)==exp (b^jl−b^10+∑i=1I∑m=0M(xi)(w^im,jl−w^im,10)x˜im(n))∑j′=1Jlexp (b^j′l−b^10+∑i=1I∑m=0M(xi)(w^im,j′l−w^im,10)x˜im(n)) ,

if $\tilde{z}(n)=l$ and 0 otherwise. Then, if we use the definition of the generative model in Equation 5, we can write both the numerator and denominator as:

(23) $$p_{\left(n\right)}(a_{j}^{l}=1)==\frac{p(\text{a}=(l,j),\overset{˜}{x}(n),\overset{˜}{z}(n);\theta )/p(\text{a}=(0,1),\overset{˜}{x}(n);\theta )}{\overset{J^{l}}{\underset{j\prime =1}{\sum }}p(\text{a}=(l,j\prime ),\overset{˜}{x}(n),\overset{˜}{z}(n);\theta )/p(\text{a}=(0,1),\overset{˜}{x}(n);\theta )}\;.$$

By multiplying now both the numerator and denominator by $p(\text{a}=(0,1),\tilde{x}(n),\tilde{z}(n);\theta )$ and by using the fact that:

(24) $$\overset{J^{l}}{\underset{j\prime =1}{\sum }}p(\text{a}=(l,j\prime ),\overset{˜}{x}(n),\overset{˜}{z}(n);\theta )=p(\overset{˜}{x}(n),\overset{˜}{z}(n);\theta )\;,$$

we finally arrive at:

(25) $$p_{\left(n\right)}(a_{j}^{l}=1)=p(\text{a}=(l,j)|\overset{˜}{x}(n),\overset{˜}{z}(n);\theta )\;.$$

Hence, the neurons α sample from $p(\text{a}|\overset{˜}{x}(n),\tilde{z}(n);\theta )$. As we pointed out at the beginning, this derivation is an important step in the proof that the plasticity rules implement the Expectation Maximization algorithm (see next subsection).

#### The plasticity rules minimize the KL divergence through Expectation Maximization

Here we further discuss the key property of the learning module pointed out in Results, section “A network module for learning stochastic associations,” that the plasticity rules modify the synaptic weights and biases in a way that minimizes the KL divergence between the full joint distribution of the generative model p(z,x; θ) and the corresponding target joint distribution $p^{*}(z,x)$. We denote the KL divergence between p(z,x; θ) and $p^{*}(z,x)$ as the objective function U(θ) ie,

(26) $$U(\theta )=D_{KL}\left(p^{*}(z,x)\left|\right|p(z,x;\theta )\right)\;.$$

By using Equation 26, the key property of the learning module can be reformulated in form of the following theorem:

**Theorem 1.** The synaptic and intrinsic plasticity rules introduced in Equations 1, 2, and 3 change the parameters θ so that they always converge to a local minimum of U(θ) subject to the normalization constraints (Eq. 6).

The complete proof of the theorem is given in the next section “Proof that the plasticity rules minimize the objective function U(θ) through Expectation Maximization.” In this section we discuss the approach and the main steps of the proof, and we also describe how the convergence is carried out through the EM algorithm, ie, how the firing of the neurons and the synaptic weight updates by the plasticity rules implement different steps of EM. The complete details to the EM implementation can be found in the section “Proof that the plasticity rules minimize the objective function U(θ) through Expectation Maximization.”

The proof of Theorem 1 uses results from the Robbins–Monro methods for stochastic approximation (Kushner and Yin, 2003). What is shown in the proof is that first, STDP and intrinsic plasticity drive the parameters θ toward satisfying the normalization constraints in Equation 6. As a second step, it is shown that given that θ satisfy the normalization constraints, then the mean update of the parameters averaged over many presented examples is in the direction of the negative gradient of the objective function U(θ). From these two intermediate steps, it follows that the parameters converge to a local minimum of U(θ) under the normalization constraints.

By reducing the KL divergence between $p^{*}(z,x)$ and p(z,x; θ), the module also aims to approximate the target conditional $p^{*}(z|x)$ by its internal model conditional p(z|x; θ) that defines the firing of the output neurons. In particular, the objective function U(θ) is an upper bound of the function

(27) $$J(\theta )={〈D_{KL}(p^{*}(z|x)||p(z|x;\theta ))〉}_{p^{*}(x)}\;,$$

which is the mean KL divergence between the target conditional and the model conditional, averaged over the distribution p^*^(**x**) of the inputs. This is easy to see from the equality:

(28) $$U(\theta )=J(\theta )+D_{KL}(p^{*}(x)\left|\right|p(x;\theta ))\;.$$

Thus, EM aims to reduce the measure of how much the two conditionals differ J(θ) through minimizing its upper bound U(θ). If the upper bound during learning decreases, this does not always mean J(θ) will decrease as well. Nevertheless, if the upper bound U(θ) converged during learning to a certain small value *C*, then after learning J(θ)≤C. And if *C* is small enough so that p(z|x; θ) is a good approximation of $p^{*}(z|x)$, in such a way the minimization of the upper bound would lead to good learning of the conditional. Furthermore, in the simulation experiments we showed that the learning based on minimizing the upper bound U(θ) works well.

The update of the synaptic weights and the biases of the neurons in the WTA circuit can be understood as performing an online stochastic approximation of the EM algorithm. The EM algorithm is an iterative optimization algorithm that finds a local minimum of U(θ) indirectly through another function called the complete data log-likelihood. In order to establish the link with the neural network learning, we will consider a stochastic version of the EM algorithm (Wei and Tanner, 1990; Jank, 2006), that we describe in the following. We will also assume the more common version used in the literature where we have a finite sequence of examples $〈\tilde{z}(n),\tilde{x}(n)〉$ for *n* = 1, . . . , *N*, instead of an infinite sequence drawn from $p^{*}(z,x)$. In the case of a finite sequence, the target distribution $p^{*}(z,x)$ is defined as the histogram of all examples. The main idea in EM is that we can substitute the original learning objective: to update the mixture generative model p(z,x; θ) so that it gets close to $p^{*}(z,x)$ by another learning objective: to update the full (complete data) generative model p(a,x,z; θ) (together with the hidden RVs a) to get closer to the complete data target distribution:

(29) $$p^{*}(\text{a},x,z;\theta )=p(\text{a}|x,z;\theta )p^{*}(x,z)\;.$$

As there are not any complete data samples to learn the generative model p(a,x,z; θ), but just incomplete data examples for the RVs **x**, *z*, in stochastic EM one completes the data samples by generating sample values for the hidden RVs a from the generative model itself, ie, through the conditional distribution p(a|x,z; θ). This is done for all examples $\tilde{x}(n)$ for *n* = 1, . . . , *N*. Then one updates the parameters so that the likelihood that the complete data examples were generated by the complete data generative model p(a,x,z; θ) is increased. The completion of the data samples is called the “expectation” step, whereas the update of the parameters of the complete data generative model to increase the likelihood of the complete data examples is called the “maximization” step. The EM optimization is performed iteratively, where in each iteration one repeats these two steps. We can link the above two steps in the EM algorithm to concrete mechanisms in the dynamics of the WTA circuit during learning. Specifically, the expectation step is implemented through the firing of spikes by the neurons α during the presentation of the data examples $〈\overset{˜}{x}(n),\tilde{z}(n)〉$. Indeed, the firing of α generates samples from the conditional distribution p(a|x,z; θ) (see section “Deriving the probability distribution of the firing of the neurons α”). The presented data examples together with the generated samples by the neurons α form complete data samples from p^*^ in Equation 29. The plasticity rules update the synaptic weights and biases exactly based on these complete data samples encoded in the spikes. Furthermore, given that the normalization constraints hold, the mean update of the parameters over the infinite sequence of examples (where the same finite sequence is repeated in succession infinitely) is in the direction of the gradient of the complete data log-likelihood (for details see section “Proof that the plasticity rules minimize the objective function U(θ) through Expectation Maximization”). In view of this established link between the offline mean update of the weights and the EM algorithm, we see that the online plasticity rules in the neural network implement an online stochastic approximation of the EM algorithm in the spirit of Monte Carlo EM (Wei and Tanner, 1990; Jank, 2006).

During learning of the distribution $p^{*}(x|z=l)$ by the WTA circuit $\alpha^{l}$, the neurons in $\alpha^{l}$ self-organize so that each of them specializes to fire in response to one cluster of similar, frequently occurring input patterns. In the probability distribution $p^{*}(x|z=l)$, this cluster corresponds to one mode of the distribution. A mode of the distribution can be described as a region of high probability in the probability space, surrounded by regions of low probability. The theoretical basis of the learning strategy via EM proves that the plasticity rules change the synaptic weights and biases to reduce the difference between the distribution of the inputs $p^{*}(x|z=l)$ and the represented generative model p(x|z=l; θ). At the end, the learning process yields a mixture distribution where each neuron in $\alpha^{l}$ represents one mixture component in the form of the unimodal distribution $p(x,a_{j}^{l}=1;\theta )$ centered at one of the clusters of input patterns. The full generative model p(x|z=l; θ) is then retrieved as a sum of the unimodal distributions. The unimodal mixture component is implicitly represented in the weights and the bias of the corresponding neuron, where the vector of synaptic weights of the neuron actually represents the center of the mixture component, ie, the location in the input pattern space where its mode peaks with maximum probability.

### Proof that the plasticity rules minimize the objective function U(θ) through Expectation Maximization

We will give in this subsection a proof of Theorem 1 from the previous subsection “The plasticity rules minimize the KL divergence through Expectation Maximization.” The theorem captures the main property of the learning module discussed in Results, section “A network module for learning stochastic associations,” ie, that the plasticity rules install in the module an internal representation of the stochastic associations between the variables **x** and *z* in the presented examples.

Before we present the main part of the proof, we first introduce some needed definitions and derivations.

#### Definitions and assumptions

According to the learning procedure, independent and identically distributed examples $〈\overset{˜}{x}(0),\tilde{z}(0)〉,\;〈\overset{˜}{x}(1),\tilde{z}(1)〉,\;. . .,\;〈\overset{˜}{x}(n),\tilde{z}(n)〉,\;. . .$ drawn from the target probability distribution are presented to the learning module one by one, each presented for a certain period of time Δ*t_E_*. The learning procedure was explained in section “Theoretical properties of the basic learning module (stochastic association module) and its plasticity.” The time interval Δ*t_E_* for the presentation of the examples should be several times larger than the duration of the PSPs *τ*. The reason for this is that at the beginning of a time period Δ*t_E_* when a new example $〈\overset{˜}{x}(n+1),\tilde{z}(n+1)〉$ is presented to the module, the values of the RVs **x** determined by the firing of the input neurons χ do not immediately change to the new example. Additionally, the neurons α do not immediately start to sample from the new conditional distribution $p(\text{a}|\overset{˜}{x}(n+1),\tilde{z}(n+1);\theta )$. There can be residual active EPSPs at the synaptic inputs connecting from the input neurons that encode the values $\overset{˜}{x}(n)$ from the previous time period Δ*t_E_*. Similarly, there can be residual EPSPs from active neurons in α that prevent the other α neurons to immediately start sampling correctly from the new conditional distributions $p(\text{a}|\overset{˜}{x}(n+1),\tilde{z}(n+1);\theta )$. Therefore, for a time period of duration of one EPSP *τ* at the beginning of the presentation of a new example, the update of the synaptic weights and biases might be incorrect. Nevertheless, if *τ* is several times smaller than Δ*t_E_*, this incorrect contribution to the learned weight (or bias) update should be insignificant.

As Δ*t_E_* is larger than *τ*, this means that for each example $〈\overset{˜}{x}(n),\tilde{z}(n)〉$ the WTA circuit generates several samples from the conditional $p(\text{a}|\overset{˜}{x}(n),\tilde{z}(n);\theta )$ as the neurons α spike several times during Δ*t_E_* (see section “Deriving the probability distribution of the firing of the neurons α”). For simplicity of notation and formulation, in the convergence proof we will assume that for each $\overset{˜}{x}(n)$ exactly one sample $\overset{˜}{a}(n)$ from $p(\text{a}|\overset{˜}{x}(n),\tilde{z}(n);\theta )$ is generated. The convergence proof can be easily extended for the more general case when several samples are generated from $p(\text{a}|\overset{˜}{x}(n),\tilde{z}(n);\theta )$. Given this assumption, the presented data example $〈\overset{˜}{x}(n),\tilde{z}(n)〉$ together with the generated sample $\overset{˜}{a}(n)$ by the neurons α represent one complete data example $〈\overset{˜}{x}(n),\overset{˜}{z}(n),\overset{˜}{a}(n)〉$ drawn from the complete data distribution $p^{*}(x,z,\text{a};\theta )$.

If with θ(n) we denote the parameter values before applying the learning rule for the *n*-th example, and with θ(n+1) we denote the values after the application of the learning rule, then:

(30) θ(n+1)=Ω(θ(n)+η(n)Δθ(n)) ,

where *η*(*n*) is the learning rate used in the *n*-th iteration, Δθ(n) is the update according to the learning rules defined in Equations 1, 2, and 3 (for easier reference, the synaptic and intrinsic plasticity rules are also given in Table 2), and Ω(θ) is a function that clips the parameter values within the intervals $-w_{min}\leq {\hat{w}}_{im,j}^{l}\leq 0$ and $-b_{min}\leq {\hat{b}}_{j}^{l}\leq 0$. The set of values θ that are within these intervals we will denote with D(θ). The subset of D(θ) where the normalization constraints (Eq. 6) are satisfied we will denote with $C_{\theta }$. As the update rule for the biases $b_{j}^{l}$ in Table 2 is defined in continuous time, we need to transform it into a form consistent with the previously stated simplification that the updates are performed for one data example drawn from $p^{*}(x,z,\text{a};\theta )$, or in other words, that the population of neurons α fires one spike during the presentation of a data example $〈\tilde{x}(n),\tilde{z}(n)〉$. We can do that easily by assuming that the examples are presented for a period of *τ* (corresponding to one spike sample), which yields the following update for the model parameters encoded in the biases:

(31) $$\Delta {\hat{b}}_{j}^{l}=\tau a_{j}^{l}\;\exp\;(-{\hat{b}}_{j}^{l})-\tau \;.$$

**Table 2.** Synaptic and intrinsic plasticity rules in the neural network of the basic learning module

**Synaptic plasticity**
At each postsynaptic spike of the neuron $\alpha_{j}^{l}$, at time *t*, the synaptic weight $w_{im,j}^{l}$ undergoes an update $w_{im,j}^{l}\leftarrow w_{im,j}^{l}+\eta \Delta w_{im,j}^{l}$ where *η* is the learning rate and
$\Delta w_{im,j}^{l}=\{\begin{array}{cc} e^{-(w_{im,j}^{l}+w_{-})}-1, & \text{if}\chi^{im}\text{fired in}[t-\tau ,t],\\ -1, & \text{if}\chi^{im}\text{did not fire in}[t-\tau ,t]. \end{array}$
The parameter *w*_–_ is a baseline parameter, and *τ* is a parameter that corresponds to the duration of PSPs.
**Intrinsic plasticity**
At the time of each spike of the neuron $\alpha_{j}^{l}$ the bias instantaneously changes its value according to $b_{j}^{l}\leftarrow b_{j}^{l}+\eta \prime \Delta b_{j}^{l}$, where
$\Delta b_{j}^{l}=\tau e^{-(b_{j}^{l}+b_{-})},$
η′ is the learning rate and $b_{-}$ is a baseline parameter for the bias. In addition, between spikes the bias exhibits continuous decay according to the differential equation ${\dot{b}}_{j}^{l}=-\eta \prime .$

Although in Table 2 the learning rates are different for the weights and the biases, here for simplicity we assume that the learning rate is the same for all parameters. We additionally assume that the sequence of learning rates η(n) satisfies:

(33) $$\begin{array}{cc} \overset{\infty }{\underset{n=1}{\sum }}\eta (n) & =\;\infty \;\text{ and }\\ \overset{\infty }{\underset{n=1}{\sum }}\eta {\left(n\right)}^{2} & <\;\infty \;. \end{array}$$

The initial values of the parameters θ(0) are randomly drawn from D(θ).

As pointed out previously (see section “Theoretical properties of the basic learning module (stochastic association module) and its plasticity”), in the joint distribution $p^{*}(x,z)$ of the examples zero values of *z* and *x^i^* do not occur, ie, $p^{*}(x^{i}=0)=0$ for all *i* = 1, . . . , *I*, and additionally $p^{*}(z=0)=0$. In the implicitly represented generative model p(x,z; θ) the probabilities where at least one of the RVs *z* and *x^i^* (*i* = 1, . . . , *I*) has zero value are represented by the fixed parameters (see Eq. 11 and accompanying text) and are not learned. The fixed parameters assume values (see Eq. 12) so that the corresponding probabilities over zero values are very small, ie, close to zero. Ideally, for the theoretical analysis we set these parameters so that:

(34) $$\begin{array}{ll} & p(x^{i}=0;\theta )=0\text{ for all }i\in \{1,\;.\;.\;.\;,I\},\text{ and }\\ & p(z=0;\theta )=0\;, \end{array}$$

in order to match the values p^*^(*x^i^* = 0) = 0 and p^*^(*z* = 0) = 0. In simulations, the probabilities over zero values represent a very small, insignificant portion of the mass of the learned generative model. This is also true when learning with a network of interconnected learning modules (see section “Theoretical properties of networks of recursively interconnected basic learning modules”).

The assumption (Eq. 34) can be easily achieved by letting in (Eq. 12) the constant *V* to converge to *V* → +*∞*. Indeed, from *V* → +*∞* it follows that w^i0,jl→−∞ and b^10→−∞. The weight offset *w*_–_ remains constant at any time during the limit process (Eqs. 12, 13). The offset value for the biases does not remain constant, however, it converges to $b_{-}\to -\infty$. To emulate this idealized condition in simulations it is sufficient to set *b*_–_ to a very large negative value. This ensures that the biases of the active α neurons converge to large positive values, which then entails that there is always a non-empty subset of α neurons that have high firing rates upon presentation of an example (see section “Deriving the probability distribution of the firing of the neurons α”). High firing rates of the α neurons imply p(a=(0,1)|x; θ)≈0 and consequently p(z=0; θ)≈0 in Equation 34. In the section “Theoretical properties of networks of recursively interconnected basic learning modules,” we also demonstrate that after the convergence of the weights and biases during learning to a local optimum, large negative value of *b*_–_ implies p(z=0|x; θ)≈0, which is consistent with Equation 34.

#### Finding a local minimum of U(θ) through Expectation Maximization

We describe here the EM algorithm applied to the concrete case of finding a local minimum of the objective function U(θ) in Equation 26, without any reference to the learning module and how it is implemented there. After we give the convergence proof that the plasticity rules drive the synaptic weights and biases to a local minimum of U(θ), we return to the question what mechanisms in the dynamics of the learning module implement different steps of the EM algorithm.

The objective function U(θ) can be written as follows:

(35) $$U(\theta )=-L(\theta )-S_{p^{*}}(x,z)\;,$$

where

(36) $$\begin{array}{c} L(\theta )=E_{p^{*}(x,z)}\left[\log\;p(x,z;\theta )\right] \end{array}$$

is the log-likelihood function and

(37) $$S_{p^{*}}(x,z)=-\sum_{x,z}p^{*}(x,z)\;\log\;p^{*}(x,z)$$

is the entropy of the RVs **x** and *z* with respect to the target joint distribution $p^{*}(x,z)$. As $S_{p^{*}}(x,z)$ does not depend on the parameters θ, the constrained local minima of U(θ) match the constrained local maxima of L(θ).

In the following we will explain the steps of the EM algorithm that finds a local maximum of L(θ). EM optimizes L(θ) indirectly through another function called the complete data log-likelihood

(38) $$\overset{˜}{L}(\theta )=E_{p^{*}(\text{a},x,z;\theta )}\left[\log\;p(\text{a},x,z;\theta )\right]\;,$$

where $p^{*}(\text{a},x,z;\theta )$ is the complete data probability distribution in Equation 29. It is an algorithm that updates the parameters θ in iterations. We will denote the values of the parameters in current iteration as $\theta_{old}$. Let $Q(\theta ,\theta_{old})$ be the complete data log-likelihood $\overset{˜}{L}(\theta )$, where in the probability distribution in the expectation we have the parameter values $\theta_{old}$ from the current iteration, ie,

(39) $$Q(\theta ,\theta_{old})=E_{p^{*}(\text{a},x,z;\theta_{old})}\left[\log\;p(\text{a},x,z;\theta )\right]\;.$$

The iteration in the EM algorithm consists of two steps:

**Expectation step:** calculate $Q(\theta ,\theta_{old})$ as the expectation in Equation 39.

**Maximization step:** find parameter values $\theta_{new}$ that satisfy:

(40) $$Q(\theta_{new},\theta_{old})>Q(\theta_{old},\theta_{old}),$$

and then set $\theta_{old}\leftarrow \theta_{new}$.

This is a formulation of EM called the generalized EM algorithm (Dempster et al., 1977). The essence of the algorithm lies in the fact that Equation 40 implies also that:

(41) $$L(\theta_{new})>L(\theta_{old}),$$

which means that in each iteration we increase the value of the log-likelihood. In fact, it also holds that:

(42) $$\frac{\partial Q(\theta ,\theta_{old})}{\partial \theta }{|}_{{\theta =\theta }_{old}}=E_{p^{*}(\text{a},x,z;\theta_{old})}\left[\frac{\partial }{\partial \theta }\log\;p(\text{a},x,z;\theta )\right]=\frac{\partial L(\theta )}{\partial \theta }{|}_{{\theta =\theta }_{old}}.$$

We will refer to this equation when we show that the learning module implements an online stochastic approximation of the generalized EM algorithm outlined here (see text after the convergence proof).

#### Definition of an auxiliary function K(θ) used in the convergence proof

We define here an additional function K(θ), which will be used in the convergence proof. A useful property of K(θ) is that within the parameter domain where the normalization constraints (Eq. 6) are satisfied, it is equal to the log-likelihood function L(θ). Hence, in order to demonstrate in the proof that the plasticity rules minimize U(θ), it will be sufficient to show that the rules drive the weights and biases to satisfy the normalization constraints, and when the normalization constraints are satisfied, they maximize K(θ).

The K(θ) function is defined as follows:

(43) $$K(\theta )=E_{p^{*}(x,z)}\left[\log\;q(x,z;\theta )\right],$$

where q(x,z; θ) is the marginal of the probability distribution:

(44) q(x,z,a; θ)==p(z|a)exp (∑i,m∑l,jw^im,jl xim ajl+∑l,jb^jl ajl)A^0(θ)∏l,j[A^ijl(θ)]ajl .

The numerator of this distribution is the same as in the definition of the generative model p(x,z,a; θ) in Equation 5. The difference in the definition between *p* and *q* is the denominator. In the denominator in Equation 44, ${\hat{A}}_{0}(\theta )$ is defined as:

(45) $${\hat{A}}_{0}(\theta )=\sum_{l}\sum_{j}\exp\;({\hat{b}}_{j}^{l})\;,$$

whereas

(46) $${\hat{A}}_{ij}^{l}(\theta )=\sum_{m}\exp\;({\hat{w}}_{im,j}^{l})\;\text{ for all }i\in \{1,. . .,I\},j\in \{1,. . .,J^{l}\}\text{ and }l\in \{0,. . .,L\}\;.$$

It can be easily seen that if the normalization constraints (Eq. 6) are satisfied, ie, if $\theta \in C_{\theta }$, then

(47) q(x,z,a; θ)=p(x,z,a; θ) ,

from which it follows that:

(48) $$K(\theta )=L(\theta )\;\text{ for all }\theta \in C_{\theta }\;.$$

The derivatives of K(θ) with respect to the parameters ${\hat{w}}_{im,j}^{l}$ are:

(49) $$\frac{\partial K(\theta )}{\partial {\hat{w}}_{im,j}^{l}}=E_{q^{*}(x,z,\text{a};\theta )}\left[\frac{\partial }{\partial {\hat{w}}_{im,j}^{l}}\log\;q(x,z,\text{a};\theta )\right]$$

where $q^{*}(x,z,\text{a};\theta )$ is

(50) $$q^{*}(x,z,\text{a};\theta )=p^{*}(x,z)\;q(\text{a}|x,z;\theta )\;.$$

If we now substitute q(x,z,a; θ) in the term inside the expectation in Equation 49 with Equation 44 and simplify, we obtain:

(51) $$\frac{\partial K(\theta )}{\partial {\hat{w}}_{im,j}^{l}}=E_{q^{*}(x,z,\text{a};\theta )}\left[x^{im}a_{j}^{l}\right]-E_{q^{*}(x,z,\text{a};\theta )}\left[a_{j}^{l}\right]\exp\;({\hat{w}}_{im,j}^{l})\frac{1}{{\hat{A}}_{ij}^{l}(\theta )}\;.$$

Similarly, for the derivative of K(θ) with respect to ${\hat{b}}_{j}^{l}$ we have the following:

(52) $$\frac{\partial K(\theta )}{\partial {\hat{b}}_{j}^{l}}=E_{q^{*}(x,z,\text{a};\theta )}\left[\frac{\partial }{\partial {\hat{b}}_{j}^{l}}\log\;q(x,z,\text{a};\theta )\right]\;,$$

and if we substitute Equation 44, we arrive at:

(53) $$\frac{\partial K(\theta )}{\partial {\hat{b}}_{j}^{l}}=E_{q^{*}(x,z,\text{a};\theta )}\left[a_{j}^{l}\right]-\exp\;({\hat{b}}_{j}^{l})\frac{1}{{\hat{A}}_{0}(\theta )}\;.$$

#### Deriving the local minima of U(θ) under the constraints

As we stated previously, the constrained local minima of U(θ) match the constrained local maxima of the log-likelihood L(θ). Additionally, the constrained local maxima L(θ) also match the constrained local maxima of K(θ), because L(θ)=K(θ) for all $\theta \in C_{\theta }$. Here we derive the local maxima of K(θ) under the normalization constraints in Equation 6in analytical form. The result is used in the main proof in the subsection “The convergence theorem and the proof” where we will show that the sequence θ(n) during learning converges exactly to these local maxima.

The local maxima of K(θ) can be found by calculating the derivatives of the Lagrangian function of K(θ):

(54) $$\Lambda (\theta ,\lambda )=K(\theta )+\lambda_{0}\left(\sum_{l=1}^{L}\sum_{j}\exp\;({\hat{b}}_{j}^{l})-1\right)++\sum_{l=1}^{L}\sum_{j,i}\lambda_{ij}^{l}\left(\sum_{m=1}^{M(x^{i})}\exp\;({\hat{w}}_{im,j}^{l})-1\right)\;.$$

The set of constrained local maxima is a subset of the solutions of the following equations obtained by setting the derivatives of the Lagrangian to 0:

(55) ∂Λ∂b^jl=Eq*(x,z,a; θ)[ajl]+(λ0−1) exp (b^jl)=0   for all l≠0 and j, and ∂Λ∂w^im,jl = Eq*(x,z,a; θ)[ximajl] ++ (λijl−Eq*(x,z,a; θ)[ajl]) exp (w^im,jl)=0    for all l≠0,j and m≠0 .

By summing the left and right sides of all equations in which *λ*_0_ occurs, and by using the equalities $\sum_{l\neq 0,j}a_{j}^{l}=1$ and $\sum_{l\neq 0,j}\exp\;({\hat{b}}_{j}^{l})=1$, we obtain a solution for the Lagrange multiplier equal to

(56) $$\lambda_{0}=0\;.$$

Similarly, if we sum the left and right sides of all equations where $\lambda_{ij}^{l}$ occurs, and if we use the equalities $\sum_{m\neq 0}x^{im}=1$ and $\sum_{m\neq 0}\exp\;(w_{im,j}^{l})=1$, we arrive at:

(57) $$\lambda_{ij}^{l}=0\;\text{ for all }l\neq 0\text{, }i\text{ and }j\;.$$

Interestingly, as all Lagrangian multipliers are 0, the critical points of the Lagrangian are also critical points of K(θ), which means that the constrained local maxima of K(θ) are also its unconstrained local maxima.

Based on these solutions for the Lagrange multipliers, we get from Equation 55 the following implicit solutions for the parameters ${\hat{w}}_{im,j}^{l}$ and ${\hat{b}}_{j}^{l}$:

(58) b^jl = log Eq*(x,z,a; θ)[ajl]=log q*(ajl=1; θ)  and w^im,jl = log Eq*(x,z,a; θ)[ximajl]Eq*(x,z,a; θ)[ajl]=log q*(xim=1|ajl=1; θ) ,

where $q^{*}(a_{j}^{l};\theta )$ and $q^{*}(x^{im}|a_{j}^{l};\theta )$ are marginal and conditional distributions derived from $q^{*}(x,z,\text{a};\theta )$ in Equation 50. Note that these are implicit solutions, since $q^{*}(a_{j}^{l};\theta )$ and $q^{*}(x^{im}|a_{j}^{l};\theta )$ depend on the parameters θ. It can be easily verified that the solutions of Equation 58 fulfill the normalization constraints. Indeed:

(59) $$\sum_{l,j}\exp\;({\hat{b}}_{j}^{l})=\sum_{l\neq 0,j}q^{*}(a_{j}^{l}=1;\theta )=1\;,$$

and also:

(60) $$\sum_{m}\exp\;({\hat{w}}_{im,j}^{l})=\sum_{m\neq 0}q^{*}(x^{im}=1|a_{j}^{l}=1;\theta )=1\;.$$

Therefore, we can substitute in the implicit solutions the probability distribution $q^{*}(x,z,a;\theta )$ with $p^{*}(x,z,a;\theta )$ from (29), which yields:

(61) $$\begin{array}{c} {\hat{b}}_{j}^{l}\;=\;\log\;p^{*}(a_{j}^{l}=1;\theta )\;\text{ and }\\ {\hat{w}}_{im,j}^{l}\;=\;\log\;p^{*}(x^{im}=1|a_{j}^{l}=1;\theta )\;. \end{array}$$

We will denote the set of all finite points θ that satisfy Equation 61 as *G*. It is assumed that the bounds of the parameters *w_min_* and *b_min_* are chosen so that G∈D(θ). The critical points of the Lagrangian in *G* are either local maxima, local minima, or saddle points of the objective function U(θ) in Equation 26under the normalization constraints in Equation 6.

#### The convergence theorem and the proof

After introducing the necessary definitions and notations in the previous subsections, we use them here to restate the Theorem 1 from the subsection “The plasticity rules minimize the KL divergence through Expectation Maximization” in more technical terms.

Theorem 1^*^. The infinite sequence θ(n) in Equation 30 converges with probability 1 to the set of local minima of U(θ) in Equation 26 subject to the normalization constraints (Eq. 6).

**Proof:** We define a “mean limit” ordinary differential equation (ODE) that corresponds to the sequence θ(n) as $\overset{˙}{\theta }=\overset{¯}{h}(\theta )$, where $\overset{¯}{h}(\theta )=E_{p^{*}(x,z,\text{a};\theta )}[\Delta \theta ]$. Here Δθ is defined with the plasticity rules in Table 2. As for all *n* the parameters θ(n) remain in the bounded set D(θ), it follows that $\underset{n}{\sup\;}E_{p^{*}(x,z,\text{a};\theta )}[||\Delta \theta ||]<\infty$ and $\underset{n}{\sup\;}E_{p^{*}(x,z,\text{a};\theta )}[{\left(\Delta \theta \right)}^{2}]<\infty$.

In order to prove convergence, we use the stochastic convergence Theorem 3.1 in Chapter 5 from Kushner and Yin, (2003) which states that, under the above assumptions, the sequence θ(0),θ(1), . . . ,θ(n), . . .  from Equation 30 converges with probability 1 to the stable points of the limit set of the mean limit ODE for all initial conditions θ(0)∈D(θ). The limit set of the mean limit ODE with respect to a set of the initial conditions θ(0)∈A, which we denote as *F*(*A*), is defined as:

(62) $$F(A)=\underset{s\to \infty }{\lim\;}\;\;\underset{\theta \prime \in A}{\cup }\left\{\theta (s\prime ),s\prime \geq s\;:\;\theta (0)=\theta \prime \right\}\;.$$

Convergence to the limit set *F*(*A*) with probability 1 means that for all stochastic realizations of the sequence θ(n) it holds that:

(63) $$\underset{n\to \infty }{\lim\;}\underset{{}_{\theta \in F(A)}}{\min\;}|\theta (n)-\theta |=0\;.$$

The main part of the proof will be to show that F(D(θ))=G, ie, that the limit set of the ODE with the set of initial values D(θ) is equal to the critical points of the Lagrangian Λ(θ,λ), which are also critical points of K(θ). We will show that in two steps. First, we will show that all trajectories of the ODE asymptotically converge to the set $C_{\theta }$ where the normalization constraints (Eq. 6) are satisfied. Then in the second step, we will show that all trajectories with initial conditions $\theta (0)\in C_{\theta }$ converge to *G*.

Let $c_{0}(\theta )$ and $c_{ij}^{l}(\theta )$ be the functions that express how much the sums in the normalization constraints (Eq. 6) deviate from 1, ie:

(64) c0(θ)=∑l∑jexp (b^jl)−1cijl(θ)=∑mexp (w^im,jl)−1 .

For all points θ in the set $C_{\theta }$, where the constraints are satisfied, we have $c_{0}(\theta )=0$ and $c_{ij}^{l}(\theta )=0$ (for all *l*≠0 and all *i* and *j*). We now analyze how the values of $c_{0}(\theta )$ and $c_{ij}^{l}(\theta )$ change when θ progresses along a solution trajectory of the mean limit ODE. For that purpose we calculate the dot products between $\overset{¯}{h}(\theta )$ and the gradients of $c_{0}(\theta )$ and $c_{ij}^{l}(\theta )$. The non-zero components of the gradient of $c_{0}(\theta )$ are:

(65) $$\frac{\partial c_{0}}{\partial {\hat{b}}_{j}^{l}}=\exp\;({\hat{b}}_{j}^{l})\;.$$

Similarly the gradient of $c_{ij}^{l}(\theta )$ has the following non-zero components:

(66) $$\frac{\partial c_{ij}^{l}}{\partial {\hat{w}}_{im,j}^{l}}=\exp\;({\hat{w}}_{im,j}^{l})\;.$$

Thus, for the dot products we have:

(67) h¯(θ)·dc0dθ=∑l≠0∑j(Ep*(x,z,a; θ)[ajl] exp (−b^jl)−1) exp (b^jl)==−c0(θ) ,

and

(68) h¯(θ)·dcijldθ=∑m≠0(Ep*(x,z,a; θ)[ajlxim] exp (−w^im,jl) −− Ep*(x,z,a; θ)[ajl]) exp (w^im,jl)=−p*(ajl=1;  θ) cijl(θ) , 

where $p^{*}(a_{j}^{l}=1;\theta )$ is a marginal of $p^{*}(x,z,\text{a};\theta )$. From these equations we can conclude that $\underset{s\to \infty }{\lim\;}c_{0}(\theta (s))=0$ and $\underset{s\to \infty }{\lim\;}c_{ij}^{l}(\theta (s))=0$ for all l≠0 and all *i* and *j*. Thus, because $\overset{¯}{h}(\theta )$ is continuous and differentiable in D(θ), it follows that the limit set of the ODE for initial conditions $\theta (0)\in D(\theta )\backslash C_{\theta }$ is inside $C_{\theta }$, ie, $F(D(\theta )\backslash C_{\theta })\subseteq C_{\theta }$. Additionally, the differentiability and continuity of $\overset{¯}{h}(\theta )$ imply that F(A)=F(F(A)) and $F(A_{1})\subseteq F(A_{2})$ if $A_{1}\subseteq A_{2}$, for all $A,A_{1},A_{2}\subseteq D(\theta )$. By using these properties, we can derive that:

(69) $$F(D(\theta )\backslash C_{\theta })=F(F(D(\theta )\backslash C_{\theta }))\subseteq F(D(\theta )\cap C_{\theta }).$$

We continue now with the second step of the proof where we show that trajectories starting within $C_{\theta }$ converge to the critical points of the Lagrangian Λ(θ,λ). First, from Equations 67 and 68, it follows that $\overset{¯}{h}(\theta )\cdot \frac{dc_{0}}{d\theta }=0$ and $\overset{¯}{h}(\theta )\cdot \frac{dc_{ij}^{l}}{d\theta }=0$ for all $\theta \in C_{\theta }$. This means that trajectories θ(s) with $\theta (0)\in C_{\theta }$ remain in $C_{\theta }$, ie, $\theta (s)\in C_{\theta }$ for all s≠0. Furthermore, it is easy to show that the set of critical points *G* of the Lagrangian Λ(θ,λ) written in implicit form in Equation 61is the same as the set of the stationary points of the mean limit ODE obtained as a solution of the equation $\overset{˙}{\theta }=0$. What remains to be shown is that the trajectory always converges with probability 1 to one of the stationary points in *G*, ie, that for example it does not enter a limit cycle. We do that by analyzing how the function K(θ) changes along the trajectory of the ODE by calculating the dot product between $\overset{¯}{h}(\theta )$ and the gradient $\frac{\partial K(\theta )}{\partial \theta }$ given in Equations 51 and 53. As for $\theta \in C_{\theta }$ it holds that $\hat{A}(\theta )=1$ and ${\hat{A}}_{ij}^{l}(\theta )=1$, the dot product is:

(70) h¯(θ) · ∂K(θ)∂θ=     =∑l≠0∑jexp (−b^jl)(Ep*(x,z,a; θ)[ajl]−exp (b^jl))2 + +∑l≠0∑j∑m≠0exp (−w^im,jl)(Ep*(x,z,a; θ)[ximajl−− exp (w^im,jl) ajl])2 ≥0 ,

and becomes equal to zero for the set of stationary points $\overset{˙}{\theta }=0$ which is the same as the set *G*. Hence, the ODE trajectories do not have any limit cycles and always converge to the critical points of the Lagrangian *G*, ie, $F(D(\theta )\cap C_{\theta })=G$. Together with the conclusion (Eq. 69) of the first step, we finally obtain that the limit set of the ODE is:

(71) $$F(D(\theta ))=F(D(\theta )\backslash C_{\theta })\cup F(D(\theta )\cap C_{\theta })\;==F(D(\theta )\cap C_{\theta })=G.$$

According to the stochastic convergence Theorem 3.1 in Chapter 5 from Kushner and Yin (2003), the infinite sequence θ(n) converges to the stable points of the limit set *G*. Stochastic fluctuations would drive the sequence to escape from the unstable points in the limit set. We have shown in Equation 70 that K(θ) increases along an ODE trajectory that starts within $\theta \in C_{\theta }$. This implies that also L(θ) increases as K(θ)=L(θ) for $\theta \in C_{\theta }$. Thus, it follows that the stable points of the ODE in *G* are the local constrained maxima of L(θ), subject to the normalization constraints, which concludes the proof.

By using results from the proof, we can now more precisely relate the update of the synaptic weights and biases by the plasticity rules to the maximization step of the EM algorithm, as discussed in subsection “The plasticity rules minimize the KL divergence through Expectation Maximization.” In the WTA circuit the plasticity rules update the weights and biases online, after each spike of a neuron in α. If we instead consider an offline update where the updates are averaged over many spikes of the α neurons (theoretically infinitely many), and then afterward they are applied to the weights and biases, then this offline update of the parameter vector in the limit of infinite number of spikes would be equal to:

(72) $$\overset{¯}{h}(\theta )=E_{p^{*}(x,z,\text{a};\theta )}[\Delta \theta ].$$

The vector $\overset{¯}{h}(\theta )$ is the gradient of the ODE trajectory, which means that for a sufficiently small learning rate the offline mean update will always increase the expected log-likelihood L(θ) provided that $\theta \in C_{\theta }$. This follows from the fact that L(θ) increases along the ODE trajectory as we have shown in the proof (Equation 70 and the text afterward). Furthermore, the off-line mean update increases L(θ) by calculating an expectation over the complete data samples from the distribution (Eq. 29) which represents the expectation step of the EM algorithm. In the subsection “The plasticity rules minimize the KL divergence through Expectation Maximization,” we already showed that the spikes of the neurons α together with the presented examples represent the complete data samples drawn from Equation 29. This clarifies that the offline mean update of the plasticity rules indeed implements the maximization step of the generalized EM algorithm as defined in Equations 42 and 40. From this, it follows that the online plasticity rules implement an online stochastic approximation version of the maximization step of EM.

The proof of Theorem 1 (reformulated in Theorem 1^*^ in this subsection) completes the theory behind the learning mechanisms and learning capabilities of the learning module described in Results, section “A network module for learning stochastic associations.” In particular, it rigorously shows that the learning module learns, via the EM algorithm, an internal model of the probabilistic relations between a set of variables **x** and another variable *z* in the presented examples.

### Theoretical properties of networks of recursively interconnected basic learning modules

This section contains additional details on the learning approach with networks of recursively connected learning modules, that we described in Results, section “Recursive combinations of the basic learning module enable efficient learning of complex distributions from examples.” After presenting the learning procedure for such networks, we will formulate in this section Theorem 2 which contains the theoretical basis of learning with networks of learning modules. Theorem 2 is derived from Theorem 1 (see section “The plasticity rules minimize the KL divergence through Expectation Maximization”), and shows that the plasticity mechanisms in a network of modules minimize another objective function that pertains to the represented probabilistic model in the whole network. This minimization then leads to learning an internal model of the stochastic associations between all variables *y^k^* of the presented examples.

The population of neurons that encodes the values of the RV *y^k^*(*t*) over time *t* we denote with $\nu^{k}$ (these are the output neurons of the learning module $N^{k}$). For each non-zero value *l* of *y^k^*, there is a neuron *ν^kl^* whose firing signals this value. In particular, a spike of the neuron *ν^kl^* at time *t* sets the value of *y^k^* to *y^k^* = *l* for a time period of duration *τ*, after which the value changes to *y^k^* = 0. To ensure a valid value of *y^k^*, no other neuron in $\nu^{k}$ should fire in the time interval [*t*, *t* + *τ*). This is ensured by the lateral inhibition between the $\alpha^{k}$ neurons, which drive the $\nu^{k}$ neurons to fire.

The network learns from presented examples $\tilde{y}(0)\text{,}$$\tilde{y}(1),\;. . .,\;\tilde{y}(n),\;. . .$ drawn from the target probability distribution $p^{*}(y)$. In the examples the RV *y^k^* assumes an integer value drawn from the set $\left\{1,2,. . .,M(y^{k})\right\}$. An example is presented to the network in form of injected currents in the neurons. These injected currents are assumed to originate from external neurons. The neurons in $\nu^{k}$ are driven by the injected currents such that their firing reflects correctly the values of the RVs *y^k^* in the current example $\overset{˜}{y}(n)$. More precisely, for ${\tilde{y}}^{k}(n)=l$ the neuron *ν^kl^* receives strong positive current and fires with a high firing rate, whereas the other neurons in the population $\nu^{k}$ receive a strong negative current, which prevents them from firing. The neurons α also have currents injected during learning, that give information about the current example, exactly in the same way as explained for a single learning module (see section “Theoretical properties of the basic learning module (stochastic association module) and its plasticity”), where the presented example $〈\overset{˜}{x}(n),\overset{˜}{z}(n)〉$ is here for the learning module $N^{k}$ equal to $〈{\tilde{y}}^{B(k)}(n),{\tilde{y}}^{k}(n)〉$ (Fig. 5).

The mathematical symbols that are used in the definition of the neural network are listed in Table 3.

**Table 3.** Mathematical symbols used in the definition of the network of interconnected learning modules

**Symbols related to the RVs in the target probability distribution**	
**y**	Vector of all multinomial RVs $\left(y^{1},. . .,y^{K}\right)$ from the target probability distribution $p^{*}(y)$
*y^k^*	*k*-th multinomial RV from the vector **y**
$M(y^{k})$	The maximum integer value the multinomial RV *y^k^* can assume
$p^{*}(y)$	Target probability distribution learned by the neural network
$y^{B(k)}$	Vector of the RVs from **y** that are in the Markov blanket of the RV *y^k^*
$N^{k}$	The learning module in the network that approximates the r.h.s of the NCC in Equation 77 for the neurons $\nu^{k}$ encoding the RV *y^k^*
**Output (and input) neurons of the modules** $N^{k}$ **and their associated RVs**	
$\nu^{k}$	Population of neurons in the network that together encode the value of the RV *y^k^* through population coding
*ν^kl^*	Neuron in $\nu^{k}$ whose firing signals the value *l* of the RV *y^k^*
*y^kl^*	Binary RV that assumes value 1 if and only if $y^{k}=l$; it corresponds to the coding property of the neuron *ν^kl^*.
**Other symbols**	
*k*	Superscript index used to indicate that the symbol describes an element of the learning module $N^{k}$
$\alpha^{k},\alpha^{kl},\alpha_{j}^{kl}$, *J^kl^*, $a_{j}^{kl},{\text{a}}^{kl},{\text{a}}^{k}$	Symbols for the neurons in α and their associated RV have the same meaning as the symbols for a single learning module in Table 1; the additional superscript index *k* indicates that the element belongs to the module $N^{k}$.
$b_{j}^{kl},w_{im,j}^{kl},{\hat{w}}_{im,j}^{kl}$, ${\hat{b}}_{j}^{kl},\theta^{k}$	Synaptic weights, biases and the parameters of the generative models have the same meaning as the symbols for a single learning module in Table 1; the additional superscript *k* identifies that the element belongs to the module $N^{k}$.
$p^{k}(y^{B(k)},y^{k};\theta^{k})$	Probability distribution of the mixture generative model implicitly represented in the module $N^{k}$
θ	Vector of union of all parameters in the neural network $\theta =(\theta^{1},. . .,\theta^{k})$ from all generative models (corresponding to the learning modules $N^{k}$)

As we stated in the section “Theoretical properties of the basic learning module (stochastic association module) and its plasticity,” only the non-zero values of *z* represent meaningful states of the external environment and are learned. The probability of the zero value p(z=0|x; θ) is not learned, and converges always to a very small value during learning. This can be easily seen if we express p(z=0|x; θ) through the firing probability densities $\rho_{j}^{l}(x)$ of the α neurons given input **x**:

(73) $$p(z=0|x;\theta )=\frac{1}{1+\sum_{l=1}^{L}\sum_{j=1}^{J^{l}}\rho_{j}^{l}(x)}.$$

From the inequality $\rho_{j}^{l}(x)=\frac{1}{\tau }\;\exp\;(u_{j}^{l}(x))\geq \frac{1}{\tau }\;\exp\;(b_{j}^{l})$ it follows that:

(74) $$p(z=0|x;\theta )\leq \frac{1}{1+\sum_{l=1}^{L}\sum_{j=1}^{J^{l}}\exp\;(b_{j}^{l})}.$$

If we substitute Equation 14 for the biases and use *b*_–_ = −*V*, which follows from Equations 12 and 15, we obtain:

(75) $$p(z=0|x;\theta )\leq \frac{\exp\;(-V)}{\sum_{l=0}^{L}\sum_{j=1}^{J^{l}}\exp\;({\hat{b}}_{j}^{l})}.$$

According to the proof of Theorem 1^*^ (Eq. 67), during learning the plasticity rules drive the synaptic weights and biases of the α neurons toward satisfying the normalization constraints in Equation 6, which simplifies Equation 75 to p(z=0|x; θ)≤exp (−V). As *V* is a very large positive constant (Eq. 12 and accompanying text), we finally arrive at:

(76) p(z=0|x; θ)≈0.

Hence, we have shown that after learning, the sum of the firing probabilities of the output ζ neurons is always very high (when inhibition is not active), regardless of the current input **x**. Similarly, in case of a network of learning modules, after learning, the output $\nu^{k}$ neurons in each module in the network are in strong competition and $p^{k}(y^{k}=0|y^{B(k)};\theta )$ is very small. From this it follows that in the stochastic dynamics of the spiking neural network states where there is a RV *y^k^* with value *y^k^* = 0 occur for very short time intervals, ie, they have very small probability in the internal model distribution p(y;  θ). Note that in the examples drawn from the target probability distribution p^*^ such states are not present as the RVs *y^k^* in the examples have values from 1 to *M*(*y^k^*). The zero values occur only in the neural network dynamics and they do not refer to meaningful values of variables in the external environment.

In order to show that if each learning module $N^{k}$ learns an approximation of $p^{*}(y^{k},y^{B(k)})$ in its internal model $p^{k}(y^{k},y^{B(k)};\theta^{k})$ then the stationary distribution of the whole network is close to $p^{*}(y)$, we use as a theoretical basis the neural computability condition (NCC) from Pecevski et al., (2011) and Buesing et al. (2011). The NCC has been identified there as a sufficient condition for representing a particular distribution p^*^ as stationary distribution of a network of spiking neurons. If the NCC is satisfied, then the stochastic dynamics of the resulting network produces (after some burn-in phase, whose length depends on the choice of its initial state) spontaneously network states (samples) that are drawn according to p^*^. The network state is represented here as the vector of the RV values **y**(*t*) at time *t*, where the value of each RV *y^k^*(*t*) at time *t* is encoded in the spikes of the population of neurons $\nu^{k}$ as described above. In order to have p^*^ as a stationary distribution of network states, the NCC requires that the firing probability density of *ν^kl^* should be equal to

(77) $$\rho^{kl}(t)=\frac{1}{\tau }\cdot \frac{p^{*}\left(y^{k}=l|y^{B(k)}(t)\right)}{p^{*}\left(y^{k}=0|y^{B(k)}(t)\right)},$$

if the current value of *y^k^* is *y^k^*(*t*) = 0, and otherwise the neuron should be silent. If $p^{*}\left(y^{k}=0|y^{B(k)}(t)\right)\to 0$, as we assumed is the case in the presented examples, the NCC transforms as follows: the firing probability density of *ν^kl^* at time *t* should be proportional to $p^{*}(y^{k}=l|y^{B(k)}(t))$

(78) $$\rho^{kl}(t)∼p^{*}\left(y^{k}=l|y^{B(k)}(t)\right),$$

if *y^k^*(*t*) = 0, and should be 0 otherwise. Additionally, the firing probability densities *ρ^kl^* for all principal neurons in the population $\nu^{k}$ should be large enough so that if the spike of the last neuron was at time *t*, a new neuron fires almost immediately after *t* + *τ*, ie, the in-between time period where *y^k^*(*t*) = 0 should be very short (ideally its duration should be equal to 0).

The firing probability density of the neuron *ν^kl^* in the network is, according to Equation 19, equal to:

(79) $$\rho^{kl}(t)=\frac{1}{\tau }\cdot \frac{p^{k}(y^{k}=l|y^{B(k)}(t);\theta^{k})}{p^{k}(y^{k}=0|y^{B(k)}(t);\theta^{k})},$$

as it is one of the output neurons of the learning module $N^{k}$. In addition, as we have shown above, the probability $p^{k}(y^{k}=0|y^{B(k)}(t);\theta^{k})$ after learning has a very small value, which makes the firing probabilities $\rho^{kl}(t)$ very high. We see that Equation 79 has the same form as the firing probability (Eqs. 77 and 78) in the NCC, except that here we have the model conditional $p^{k}(y^{k}|y^{B(k)};\theta^{k})$ instead of the target conditional $p^{*}(y^{k}|y^{B(k)})$ in the NCC. Thus, as the plasticity rules during learning change the weights and biases toward reducing the difference between $p^{k}(y^{k},y^{B(k)};\theta^{k})$ and $p^{*}(y^{k},y^{B(k)})$, which is an upper bound of the difference between the corresponding conditionals (Eq. 28), this then should bring the firing probability $\rho^{kl}(t)$ from Equation 79 closer to the desired firing probability in the NCC (Eq. 78).

The objective function U^(θ) for learning in the whole network can be formulated as the sum of the objective functions U(θ) of all learning modules $N^{k}$:

(80) U^(θ)=∑kDKL(p*(yk,yB(k))|| pk(yk,yB(k); θk)).

Using U^(θ) we can state the following theorem for learning in the whole network:

**Theorem 2.** The synaptic and intrinsic plasticity rules in Equations 1, 2, and 3 change the parameters θ in a way so that they always converge to a local minimum of $\hat{U}(\theta )$ subject to the normalization constraints (Eq. 6).

Theorem 2 directly follows from Theorem 1 and from the fact that each of the KL divergences in the sum are parametrized by a separate set of parameters $\theta^{k}$. If $\hat{U}(\theta )=0$ then according to the NCC we have that $p(y;\theta )=p^{*}(y)$. If the parameters converge to a good local minimum of U^(θ) so that the learning modules in the network learn a good approximation of the firing probability densities of the NCC, then the stationary probability distribution p(y; θ) of the network would approximate well the target distribution $p^{*}(y)$.

During its spontaneous activity the neural network enters states where there is at least one RV *y^k^* with zero value. These states are not part of the target distribution p^*^. Nevertheless, as that the output neurons after learning fire with very high firing rates when not inhibited (Eqs. 73–76), the states with zero values occur only for very short time intervals and should not affect the correct Markov chain Monte Carlo sampling process of the stochastic network. Therefore, if the output neurons of the modules learn to fire approximately according to the NCC (78) and fire with very high firing rates, the distribution of the network states during spontaneous activity p(y; θ) should be a good approximation to $p^{*}(y)$, as we show in our computer simulations.

If it satisfies the NCC, the neural network cannot only generate samples from p^*^, but it can also perform via sampling probabilistic inference based on p^*^ (described in Results, section “Flexible retrieval of learnt statistical information through probabilistic inference”). In a typical probabilistic inference task evidence is provided for a subset of the RVs **y***_e_*, ie, their values are known, and one wants to calculate the posterior distribution $p^{*}(y_{s}|y_{e})$ for some of the unknown RVs **y***_s_* given the evidence. In the neural network, the evidence is presented by clamping the neurons with injected input currents. For example, if the value of the RV *y^k^* is *y^k^* = *l*, then positive current from exogenous neurons is injected in the neuron *ν^kl^* such that it fires with high firing rate, whereas all other neurons in the population $\nu^{k}$ are kept silent through a negative injected current. A basic property of the network is that when evidence is injected in it, it changes its dynamics such that the neurons for the unknown RVs **y***_s_* generate samples exactly from the posterior $p^{*}(y_{s}|y_{e})$. The posterior probabilities can then be estimated simply by counting the generated samples.

## Details to computer simulations

### Details to the computer simulation in Example 2

All computer simulations were carried out with NEVESIM, an event-based neural network simulator developed in C++ with a Python interface (Pecevski et al., 2014). The simulator NEVESIM builds on techniques developed in the simulator PCSIM (Pecevski et al., 2009). In all simulations we used the stochastic point neuron model from Buesing et al. (2011) and Pecevski et al. (2011). The parameter *τ* that defines the encoding of values of the RVs by the spikes was equal to *τ* = 15 ms. The neurons had absolute refractory period of duration *τ*. The lateral inhibition in the WTA circuits was implemented with a population of five inhibitory neurons, where all neurons in the population α connect to these inhibitory neurons with a synaptic weight *w_e_*_2_*_i_* = 80. The bias of the inhibitory neurons in the lateral inhibition was set to *b_inh_* = −10, and the bias of all neurons corresponding to the RVs **y** was also set to *b_pp_* = −10. The weights of the strong input synaptic connections to each neuron *ν^kl^*, that originated from its corresponding group of neurons in $\alpha^{kl}$ that drive it to fire, were set to *w_pp_* = 20. During learning, the evolution of all synaptic weights was confined within a range [*w_min_*, *w_max_*], where *w_min_* = 0, *w_max_* = 4 for the synapses of the α neurons in the learning modules $N^{1},\;N^{2}$ and $N^{3}$, and *w_max_* = 2 for the synapses of the α neurons of module $N^{4}$. Synaptic weight changes induced by the plasticity rule that would go below *w_min_*, or above *w_max_*, were clipped to *w_min_*, or *w_max_*, respectively.

At the beginning of each probabilistic inference simulation, the state of the network (defined by the RV values **y**(*t*)) was initialized according to a random state drawn from a uniform distribution over all possible initial network states. This was done by injecting very short current pulses in the neurons in the populations $\nu^{k}$. During the probabilistic inference simulations, evidence for the known values of the RVs was given to the network by injecting external currents in the $\nu^{k}$ neurons of the known RVs. The currents were injected in exactly the same way and with exactly the same amplitudes as during the presentation of the examples in the learning process (see below).

During learning, the presented examples were generated from the Bayesian network in Figure 6*B*, with the values for the conditional probabilities given in Table 4. The prior probabilities $p(y^{1}=2)$ and $p(y^{2}=2)$ were both equal to 0.5.

**Table 4.** Values for the conditional probabilities in the Bayesian network in Figure 6B used to generate examples for learning in Example 2

	$p^{*}(y^{3}=2|y^{1}=1,y^{2})$	$p^{*}(y^{3}=2|y^{1}=2,y^{2})$	$p^{*}(y^{4}=2|y^{2})$
*y* ^2^ = 1	0.13	0.87	0.13
*y* ^2^ = 2	0.87	0.13	0.87

During the presentation of the examples, the neuron *ν^kl^* had injected a strong positive current *I_pp_*_+_ = 30 if *y^k^* = *l* in the example, or a strong negative current *I_pp_*_–_ = −30 if in the example *y^k^* ≠ *l*. Additionally, if in the current example *y^k^* = *l*, then a strong negative external current with amplitude $I_{F}^{-}=-80$ was injected in each of the neurons in the population $\alpha^{k}$ that are not in the subpopulation $\alpha^{kl}$, whereas the neurons in $\alpha^{kl}$ did not receive any external current.

Each subpopulation $\alpha^{kl}$ in every learning module in the network consisted of two neurons. All excitatory neurons in the network had an alpha shaped EPSP defined by the kernel:

(81) $$\epsilon_{\alpha }(t)==\{\begin{array}{cc} \epsilon_{0}\cdot e\cdot (\frac{t}{\tau_{\alpha }}+t_{1})\cdot \exp\;(-(\frac{t}{\tau_{\alpha }}+t_{1}))-\frac{1}{2} & \text{ if }0<t<(t_{2}-t_{1})\tau_{\alpha },\\ 0 & \text{otherwise}\;. \end{array}$$

Here $\epsilon_{0}=2.8$ is a scaling factor, *t*_1_ and *t*_2_ and are the points in time where the basic alpha kernel of the form e·t·exp (−t) is equal to $\frac{1}{2}$, and *τ_α_* = 8.5 ms is the time constant of the alpha kernel. The same shape of the EPSPs was also used by Pecevski et al. (2011). After an incoming spike at the synapse at time $t_{1}^{f}$, the time course of the EPSP is equal to $w\epsilon_{\alpha }(t-t_{1}^{f})$ (*w* is the synaptic efficacy) until the next spike at $t_{2}^{f}$ after which it is set to $w\epsilon_{\alpha }(t-t_{2}^{f})$. The PSPs at the synapses connecting from, and to, the inhibitory neurons of the lateral inhibition had a rectangular shape with duration of *τ* = 15 ms. The rectangular IPSPs of the inhibitory neurons approximate the effect that fast-spiking bursting inhibitory neurons with short duration IPSPs would have on the membrane potential of the neurons α. The synaptic weights of the connections from the inhibitory neurons in the lateral inhibition to the neurons α were all equal to *w_i_*_2_*_e_* = −7.

Before the start of learning, the synaptic weights of all neurons in α were initialized randomly from a Gaussian distribution with mean ${\overset{¯}{w}}_{init}=w_{max}/3$ and SD *σ_w_*_0_ = 0.1. If the randomly drawn value was not in the interval [*w_min_*, *w_max_*], then it was redrawn again until the new value was in the interval (the same type of redrawing was done for the initial values of the biases). The initial biases of the α neurons were randomly drawn from a Gaussian distribution with mean ${\overset{¯}{b}}_{init}=5$ and SD *σ_b_*_0_ = 0.1. The evolution of the biases of the neurons in α was restricted to be in the range [*b_min_*, *b_max_*] with *b_min_* = –30 and *b_max_* = 5. If the intrinsic plasticity changed the bias above *b_max_* or below *b_min_*, the bias was clipped to *b_max_* or *b_min_*, respectively. In the learning rules we introduced an additional scaling factor *T* = 0.58 in the potentiation part. In particular, the potentiation part of the learning rule (Eq. 1) for the weights had the form $\Delta w=e^{-T(w+w_{-})}-1$, and the potentiation part of the learning rule (Eq. 2) for the biases had form $\Delta b=\tau e^{-T(b+b_{-})}$.

The learning process lasted 1200 s of biological time. During the first 600 s of learning, the learning rate for the synaptic weights was decreasing linearly from η=0.002 at *t* = 0 to η=0.0006 at *t* = 600 s. In the second 600 s of the learning, after *t* = 600 s, the synaptic plasticity was not active, ie, the learning rate was set to *η* = 0. The learning rate for the biases during the first 600 s was constant and equal to η′=0.01. In the second part of learning, after *t* = 600, it had another constant value equal to η′=0.02.

The offset parameter in the synaptic plasticity rule was equal to w−=2.5 log (0.2), whereas the offset parameter of the intrinsic plasticity in the neuron $\alpha_{j}^{kl}$ was:

(82) $$b_{-}^{k}=\frac{2.5}{T}\cdot (-|B(k)|\log\;(0.2)-\underset{i\in I^{B(k)}}{\sum }\log\;(M(y^{i})+1)+\log\;(0.02))\;,$$

where |B(k)| is the number of RVs in the Markov blanket of *y^k^*, **I***^B^* ^(^*^k^* ^)^ is the set of indices of the variables *y^i^* that are in the Markov blanket of *y^k^*, *T* is the scaling factor in the learning rule (see above) and *M*(*y^i^*) denotes the largest value of the RV *y^i^*. As the neurons $\alpha^{k}$ in different learning modules have different number of input synapses, the bias offset parameter was set for each neuron to counter-balance appropriately the average total input it receives from the input synapses.

In Figure 7*B*, the frequency of the network states was calculated from the spontaneous activity of the neural network after learning, simulated for 20 s biological time. The neural network was initialized to be in a random network state at *t* = 0. In Figure 8*C*, the KL divergence was calculated at 1 min time intervals, ie, at time points *t* = 60*i* seconds (*i* = 0, . . . ,19). At each time point, the distribution p(y; θ) was estimated by simulating the neural network with the values of the synaptic weights and biases of the neurons at the particular time point. The network was simulated for 1500 s, and the estimated probabilities were calculated from the network states in the time interval t∈[100s,1500s] of the simulation. In Figure 8 Panels, A, B, and D, the distributions $p^{k}(y^{k},y^{B(k)};\theta^{k})$ and $p^{k}(y^{k}|y^{B(k)};\theta^{k})$ of the learning modules were calculated analytically by using Equation 5, and then marginalizing α.

### Details to the computer simulations in Example 1

The target probability distribution $p^{*}(x^{1},x^{2},z)$ from which examples were generated is given in Table 5. The scaling factor *T* in the learning rules (see section “Details to the computer simulation in Example 2”) was set to *T* = 0.4. The maximum weight value was in this example *w_max_* = 5. Before learning, the synaptic weights of all neurons in α were initialized randomly from a Gaussian distribution with mean ${\overset{¯}{w}}_{init}=3.0$ and SD $\sigma_{w0}=0.1$. All other parameters in this example that define the learning module, as well as the learning process, were the same as the parameters of the learning modules in the computer simulation in Example 2 (see section “Details to the computer simulation in Example 2”).

**Table 5.** The target probability distribution $p^{*}(x^{1},x^{2},z)$ in Example 1

	$p^{*}(x^{1},x^{2},z=1)$	$p^{*}(x^{1},x^{2},z=2)$
*x* ^1^ = 1, *x* ^2^ = 1	0.04	0.04
*x* ^1^ = 1, *x* ^2^ = 2	0.21	0.21
*x* ^1^ = 2, *x* ^2^ = 1	0.04	0.21
*x* ^1^ = 2, *x* ^2^ = 2	0.21	0.04

## Discussion

Numerous models for probabilistic inference in networks of neurons have been proposed, and many models for the impact of learning on network computations have been proposed. However, surprisingly, these two lines of research have so far (with a few exceptions that are discussed below) not been brought together. We propose in this article a new model for learning and memory organization in recurrent networks of spiking neurons that makes the information that is gathered from numerous experiences immediately available for complex and unforeseen memory retrievals in the form of probabilistic inference. The network is able to perform such probabilistic inference just through its inherent stochastic dynamics. More precisely, we assume that the network receives samples (=examples) from some unknown multivariate distribution p^*^, and show that a suitably structured recurrent network of spiking neurons is able to build through STDP and intrinsic plasticity of the excitability of neurons an internal model for p^*^ in such a way, that it can answer a diverse repertoire of probabilistic inference queries about the stored knowledge through sampling. Early models for memory storage and retrieval in recurrent networks of artificial neurons (Steinbuch, 1961; Hopfield, 1982, 1984) had focused on the storage of isolated memory items in attractors of a deterministic network dynamics. The only memory queries that are considered in these models are input completion tasks, or finding the most similar memory item to the input pattern. Answering queries that require the combination of several stored memory items is virtually impossible in such model, especially because the stored memory items were required to be scrambled (orthogonalized) before they were committed to the network. This is inconsistent with experimental data on human memory, which require a substantially more structured memory organization (Stickgold and Walker, 2013).

We have shown in this article, that very simple network motifs (Fig. 2) provide learning modules, which can extract probabilistic relationships between random variables from examples, simply by applying STDP and intrinsic plasticity. One feature of these network motifs is that different groups of neurons on layer 2 project to different neurons on layer 3. Such nonconvergent synaptic connections are difficult to identify with current experimental methods, but have already been found in the mouse cortex (Chen et al., 2013), where largely nonoverlapping populations of pyramidal cells in layer 2/3 of area S1 project to areas S2 and M1. Somewhat similar fine-scale connectivity patterns had previously been found within a cortical column of rat visual cortex (Yoshimura et al., 2005; Kampa et al., 2006). It remains to be tested whether these fine-scale network structures in the brain support the learning of stochastic associations as predicted by our model.

These simple stochastic association modules can be recursively combined (Figs. 4, 5), and are then able to learn also complex stochastic relationships, including higher order moments (eg, explaining away), from examples. We have demonstrated this for a well-known visual inference task from Knill and Kersten (1991), which is known to require explaining away (Figs. 6–9), but where it has been an open question whether brains could in principle acquire this capability through learning. Furthermore, we have shown that this impressive learning capability of recurrent networks of spiking neurons can be understood for simple rules for STDP and intrinsic plasticity of neurons on the basis of a rigorous mathematical theory (Expectation Maximization).

The representations of statistical knowledge in networks of spiking neurons that are shown here to result from learning are structurally very similar to previously proposed ones by Pecevski et al. (2011) that were based on construction instead of learning, with one important difference: whereas the constructed networks required very large numbers of auxiliary neurons for representing random variables with larger Markov blankets, we show here that similar networks but with realistic numbers of hidden (auxiliary) neurons can still provide good approximations. These smaller modules automatically learn to make optimal use of the number of auxiliary neurons that are available to them. They learn automatically to approximate complex stochastic associations between random variables that emerge from examples, with a mixture distribution whose maximal number of components (modes) fits to the available number of auxiliary neurons. We have demonstrated this important feature of our learning approach in Figure 3 (compare panels *B*,*D* and *C*,*E*) and in our model for explaining away in Figure 6 (where the number of auxiliary neurons in the learning modules (Fig. 6*D*) is smaller than the number required by the construction of Pecevski et al.(2011)).

One other interesting general feature of our model is that it shows how noise in networks of neurons can be beneficial. In fact, noise provides here a necessary ingredient both for the self-organization of the network through STDP, and for the use of learnt probabilistic relationships for probabilistic inference via MCMC sampling (Habenschuss et al., 2013a). The necessary stochasticity in the neurons can come from different sources like for example the unreliable neurotransmitter release from the vesicles at the presynaptic site, or the stochastic closing and opening of membrane ion channels (Faisal et al., 2008).

Finally, we would like to point out that our network model produces answers to probabilistic inference queries (Fig. 9) in the form of firing rates, which is obviously useful for communicating such answers to downstream networks. Internally however, the network works with a spike-based encoding of network states, where every spike has an impact on the network state (Fig. 7).

Our model and theory has identified concrete plasticity mechanisms and network architectures that would enable networks of neurons in the brain to build probabilistic internal models for their stochastic environment. We have focused here on an idealized model in order to keep it theoretically tractable. Further work will have to explore to what extent similar learning phenomena arise in more complex neural network models that sacrifice theoretical tractability for additional biological details.

### Related work

There are several studies that propose neural implementations of probabilistic inference in general graphical models where, as in our approach, the present independencies of the distribution are directly exploited for reducing the complexity of the neural network structure. The majority of these base their implementations on the loopy belief propagation algorithm (Rao, 2006; Steimer et al., 2009; Siegelmann and Holzman, 2010; Litvak and Ullman, 2009). Except for Siegelmann and Holzman (2010), to the best of our knowledge, none of these studies proposes a way how these neural structures could emerge through learning from examples. The model of Siegelmann and Holzman (2010) is formulated on a more abstract level. It is based on the observation that belief propagation (message passing) requires only three arithmetical operations: summation, multiplication, and division (for normalization). Their network model is based on symbolic computational units (interpreted as multicompartment neurons) that carry out these arithmetical operations on real numbers. The resulting real numbers are interpreted as probabilities or messages that are sent to other units during belief propagation. Estimates of conditional probability tables are extracted from examples through online accumulators, assumed to be implemented as plasticity of the weight of a dendritic branch that represents a specific value assignment for a set of random variables (more precisely, for the Markov blanket of a random variable). Hence, this learning model is not based on synaptic plasticity, but rather on a plasticity mechanism that changes the weight of a whole dendritic branch. We are not aware of an attempt to implement this approach with spiking neurons, or with more local plasticity rules.

An alternative framework for probabilistic inference in neural circuits developed by Ma et al. (2006) and Beck et al. (2008, 2011, 2012) is based on representation of probability distributions in probabilistic population codes. To the best of our knowledge, the question of how the neural implementations in those studies can emerge through learning with local plasticity mechanisms has so far not been addressed.

We have focused in this paper on the task of learning time-invariant distributions p^*^ over static patterns. Complementary to this, in several studies (Deneve, 2008; Rezende et al., 2011; Brea et al., 2013; Kappel et al., 2014) the authors developed neural network models for learning time-varying distributions, restricted to dynamical Bayesian networks that do not have dependencies between the RVs in the same time step, which simplifies the learning. In these models, the network learns to reproduce sequences of patterns, by developing latent representations as a memory about the recent history of patterns, and learning the stochastic transitions between the patterns in the sequence. In contrast to this, the neural networks in our approach learn a probability distribution of static patterns as their stationary distribution, where the distribution can contain arbitrary dependencies between the random variables without any restrictions.

The problem of learning a probability distribution from examples has been well studied in the artificial neural network community. The Boltzmann machine is one of the earliest developed neural networks that can learn general probability distributions (Ackley et al., 1985). The learning in the Boltzmann machine is however difficult for learning higher-dimensional probability distributions with a larger number of RVs (Hinton, 2002; Carreira-Perpinan and Hinton, 2005). Building on the Boltzmann machine idea, more recently developed deep belief networks have considerably improved the efficiency and scalability of learning by using a Boltzmann machine with a two-layer bipartite graphical model structure, called restricted Boltzmann machine, which is easier to train than the general Boltzmann machine (Hinton et al., 2006). Deep belief networks learn multilayer latent representations of the input data in a generative model by training layer-by-layer restricted Boltzmann machines. As in our approach, the learned probability distribution is embodied as a stationary distribution of the network states. However, the structure of Boltzmann machines and deep belief networks exhibits symmetric weights between the neurons which are not found in biological networks of neurons, and also the biological plausibility of layerwise training and the learning rules in these networks is not clear.

These approaches derive their learning rules from the maximum likelihood learning principle. Our learning approach for network of interconnected learning modules, on the other hand, is more reminiscent to maximizing the pseudo-likelihood (Besag, 1975), an alternative parameter estimation method in statistical learning, where the objective function can be formulated through the Kullback–Leibler divergences between the target and the model conditional distributions of one variable given the rest of the RVs. The difference is that in the pseudo-likelihood objective function the conditional distributions are all derived from a single probabilistic model, whereas in our approach each learning module learns a separate generative model of a marginal of the target probability distribution.

### Experimentally testable predictions of our model and other questions for further research

Our model makes significantly different predictions compared with previous models for memory in neural networks with regard to the architecture of the neural networks involved. Models based on Hopfield networks and Boltzmann machines predict that memories are stored in homogeneous networks of only excitatory neurons with symmetric synaptic connections. In contrast, our model predicts that memories are stored in large assemblies of highly structured generic microcircuit motifs, each consisting of excitatory and inhibitory neurons. Our model gives rise to a concrete hypothesis, why different species have different learning capabilities, which cannot be explained in terms of the different numbers of neurons in their brains. It proposes that the structure or structural predisposition of interconnections between neurons is an important factor for learning performance. In particular, it predicts that the superior learning capabilities result from a genetically more precisely structured interaction of excitatory and inhibitory neurons. Our model of a probabilistic learning module (Fig. 2) proposes in fact two different functional roles of inhibitory neurons: lateral inhibition among excitatory neurons that induces each of them to specialize on different presynaptic firing patterns, and another type of inhibition that prevents hidden neurons that learn a probabilistic relationship between random variables 〈x,z〉 for a specific value *z* = *l*, to engage plasticity for examples 〈x,z〉 with *z* ≠ *l*. This architecture provides concrete hypotheses for the analysis of data on the role of inhibitory neurons in the organization of plasticity (Caroni, 2015; Letzkus et al., 2015).

Furthermore, our model for an atomic probabilistic learning module predicts that pyramidal cells within a network can represent probabilistic, rather than only deterministic relationships. This can in principle be tested experimentally by exciting (eg, through optogenetic methods) a subset *A* of these neurons, and observe the resulting firing probability of pyramidal cells *B*. Furthermore our model predicts that this firing probability of neurons in *B* can be changed upward through trials where both neurons in *A* and *B* are made to fire, and changed downward through trials where neurons in *A* are made to fire and neurons in *B* are inhibited.

In contrast to preceding memory models, our model does not predict that synaptic connections between neurons are in general symmetric. This would actually be impossible for synaptic connections between excitatory and inhibitory neurons. But also for synaptic connections between pyramidal cells in the cortex a symmetry of synaptic weights between them is not really consistent with the currently available experimental data (Song et al., 2005; Haeusler et al., 2009, their Fig. 4). With regard to the plasticity mechanisms involved, our model points to an essential contribution of intrinsic plasticity of the excitability of pyramidal cells for memory formation (Mozzachiodi and Byrne, 2010).

An interesting functional prediction of our model is that memory recall cannot only take the form of input completion (like in a Hopfield network), but can engage the full power of probabilistic inference. Furthermore, it proposes that this inference is implemented through the inherent stochastic dynamics of networks of neurons in the brain. Such implementation has previously already been proposed on the basis of data from cognitive science (Denison et al., 2013; Vul and Pashler, 2008). Furthermore our model predicts that memory recall and imagination of possible scenarios are closely related brain computations, that engage similar network mechanisms. This prediction appears to be consistent with recent experimental data, which suggest that memory recall and imagination/fabulation engage largely the same brain systems (Schacter, 2002).

Finally, our model predicts that the development of suitable brain networks that store basic insights about typical causal roles and dependencies of the objects and phenomena we encounter is essential for learning. This prediction is in line with experimental results from cognitive science, which argue that such basic knowledge about dependencies in the real world is already known to 2-year-old children (Landau et al., 1988). Because our learning approach emphasizes the role of probabilistic inference, it also provides a theoretical framework for integrating innate or previously learnt knowledge in the form of priors.

The learning paradigm that we have presented was designed to provide an alternative to other neural network models for higher-level memory. Such higher-level memory system in the brain receives high-dimensional inputs **y** from numerous brain areas, in particular also higher-level features that are extracted from sensory inputs by other learning systems.

It is at this point an open question to what extent the proposed model for learning in networks of spiking neurons can also provide insight into the organization of lower level learning systems in the brain, for example in the visual system. The underlying mathematical approach is quite general, and guarantees convergence to an internal model for any external distribution p^*^ over discrete RVs that generates the examples **y** that are presented to the network. How good this internal model will become is related to the question how well p^*^ can be approximated by a Bayesian network with small degrees of nodes, or more generally, by a simplified distribution where all RVs have small Markov blankets. However, a precise answer to this question is even more difficult, because our model is in principle also able to approximate some distributions with large Markov blankets, see the remarks at the end of Results section “Small numbers of hidden neurons in the learning modules often suffice.” Numerical tests for a number of practically relevant distributions p^*^ are likely to provide further insight into this question.

In principle, it is also possible to boost the learning capability of our approach by stacking multiple copies of the learning network. The α neurons in the network learn to encode salient combinations of values of different RVs, similarly as feature detectors on the first hidden layer of a deep learning network. Hence, it would make sense to send the output of these α neurons also as input to a version of the same type of learning network on a second level. One would then expect that the second level network learns in the same unsupervised manner to detect and represent salient combinations of values in these α neurons. The analysis of the performance of such a stacked learning architecture based on spiking neurons and STDP is a topic for future research.

### Summary

Altogether, we have shown that some forms of probabilistic inference can be learnt through STDP, even in cases where the nontrivial “explaining away” effect occurs (Figs. 6–9). We propose, that this new paradigm for network learning provides an alternative to previous models for associative learning that were based on learning categorical rather than probabilistic associations. In addition, in contrast to earlier memory models, this new model is compatible with basic properties of biological networks of neurons, such as spikes, trial-to-trial variability, stereotypical microcircuit motifs, and STDP.

## Synthesis

Tatyana Sharpee, The Salk Institute for Biological Studies The decision was a result of the Reviewing Editor Tatyana Sharpee and the peer reviewers coming together and discussing their recommendations until a consensus was reached. A fact-based synthesis statement explaining their decision and outlining what is needed to prepare a revision is listed below.

The consensus was that the concerns raised could be addressed by revising the text to clearly state the assumptions, the novel contributions of this work compared to previous ones. In addition, both Reviewers objected to the use of the term “unsupervised” to describe the approach.

Reviewer # 1:

The manuscript “Learning probabilistic inference through STDP” addresses the important and interesting problem of how networks of spiking neurons can perform probabilistic inference. Specifically, the work focuses on the the question how neural networks can learn, represent and access the probability distribution that governs the statistics of its inputs.

My main concern regarding the present form of the manuscript is the (for me) incomprehensible mixing of biological motivation, a supposedly biologically plausible implementation of an unsupervised spiking neural network, and abstract mathematics. When reading through the main text, I found it very difficult to decide if a given paragraph should be interpreted as part of a formal mathematical proof, a description of a simulation, or a justification of why a mathematical statement applies to a biological circuit in the brain. What should be made of statements like (just a small set of examples from a long list):

”In particular, we assume that in the population alpha of WTA neurons there are disjoint subpopulations alpha l for each possible value l of z, and the subpopulation alpha l projects to the zeta l neuron with strong synaptic weights so that a spike of a neuron in alpha l causes also the neuron zeta l to fire.”

or

”More precisely, we only need to assume that synaptic plasticity is blocked for these other neurons; one way of achieving this is to prevent these neurons from firing.”

or

”As there is a separate WTA population alpha l for each value l of the variable z, the hidden neurons in alpha l are able to home in on the most prominent modes of the distribution p star of x given z=l.”

or

”In order to achieve that, one just needs to allow each learning module N k to learn in parallel from those components of the example y that concern the random variables it represents in the previously described manner.”

Do these sentences refer to things that have been implemented in the simulations or are they descriptions of mathematical derivations?

The authors word strong claims, such as “universal learning capabilities” and the “virtually unlimited access to learnt information” but again it is not clear to me if these refer to mathematical idealizations or biologically plausible circuit implementations. I find the two limited “toy-examples” stand in stark contrast to these grand claims. In addition, the shown data consists almost exclusively of side-by-side plots of two probability distributions. How can the reader tell if the clear discrepancies between them are good (as the authors suggest) or bad (given the simple tasks). I would find it extremely helpful if the authors would provide a quantitative evaluation of the reported performance, ideally including a comparison to an alternative state-of-the-art approach and, with view to their claims, an analysis of how their model scales up to larger target distributions.

I also am confused by the authors choice to call their approach unsupervised (”without any supervisor or teacher”, “without any additional computational overhead or teaching signals”). If I understand the proposed implementation correctly, during learning both the input and the output of the learning circuit are specified by a given example (in addition to some other interventions that select the correct parts of the network to learn and block learning in the rest). Isn't fixing the output of a system a hallmark of supervised learning. In fact, in “What Can a Neuron Learn with Spike-Timing-Dependent Plasticity?” Legenstein et al. 2005 state: “We consider a supervised learning paradigm where during training, the output of the neuron is clamped to the target signal (teacher forcing).” I this sense I find the authors' choice to call the present learning scheme “unsupervised” misleading.

I find it impossible to reconcile the example given in the significance statement about our ability to answer complex multifaceted questions like “whether Mr. X is likely to enjoy dinner with us” with the data shown in the results. I am puzzled to what extend the authors really think that they provide a model “which can explain this capability”.

Reviewer #2:

This paper attempts to construct a neurally plausible mechanism for representing and learning probabilistic generative models. They recapitulate past work showing how to represent probability distributions as a variant of a Boltzmann machine that uses spiking neurons. Then they recapitulate past work showing how the synaptic weights in this spiking network can be learned by Spike Timing Dependent Plasticity (STDP) that implements the EM algorithm. They prove some relevant theorems about the quality of their model, and illustrate the concepts with some simple inference examples. I think the only novel aspect here is to chain past STDP learning models together into a bigger network.

Overall, I am quite sympathetic to their goals, and I think that this paper does makes an advance over past work. However, I recommend a some important clarifications, and I have a few caveats that I think are important to articulate to the target neuroscience audience. Although some of my concerns are quite serious, I do not expect all of them to be fully resolved, and so I do not necessarily view them as an insurmountable hurdle for publishing here. If the appropriate qualifications are present, readers can judge for themselves.

Also, this is a difficult paper to read. Partly this is unavoidable, because of the complicated subject matter. But partly this is because there are some confusing aspects of their presentation that can be avoided. I make some suggestions at the end, but there are many places where the language should be improved.

1) First and foremost, what is actually the new advance here? Am I correct that it is just the chaining of multiple modules together? This needs to be stated crisply and clearly.

2) The most serious objection to biological plausibility, and my biggest problem with their model, is the very strong assumption that alpha^l populations are disjoint. How would that structure be learned? This means that no two alpha neurons can project to the same zeta target. This is a major architectural constraint; how would that be learned or enforced? I don't find this reasonable. Are there any sensible ways to relax this assumption?

3) WTA implies perfect anti-correlation between neurons dedicated to different states of the same variables. This is not observed experimentally. What are the consequences of relaxing this anticorrelation?

4) A second serious empirical criticism is that firing rate coding of probabilities (as opposed to log-probabilities) is inconsistent with contrast-invariant tuning. This is because higher contrast means higher confidence, and thus narrower population responses and narrower tuning. I am not aware of data that supports this narrowing of tuning with contrast, and on the contrary there is plenty of evidence showing tuning width remains constant with contrast.

Related to this, how are we to interpret measured neural activity for neurons that are tuned to continuous variables in the world? If we have a neuron that is tuned to, e.g., orientation, then how do we identify the feature L that its responses favor? The peak? The mean? A range? In their model world, the features are clearly defined and these questions do not come up. But to relate this model to real neurons, these questions must be answered.

5) Pervasively, it is very unclear from the writing whether the learning and spiking cause the network to sample from the joint marginal or conditional distribution. Throughout, the main text seems to go back and forth on this question, and the methods are not much more helpful. I had a large number of confusions related directly or indirectly to this issue.

6) p28: “Minimizing the KL divergence between p*(z, x) and p(z, x; theta) also reduces the difference between the target conditional p*(z|x) and the model conditional p(z|x; theta).” I think this is false. Given some model constraints, one may reduce the cost function U(theta) more by reducing the KL divergence between p*(x) and p(x;theta) than by reducing J(theta), which is the average KL between p*(z|x) and p(z|x,theta). Whether this happens should depend on how the models are parameterized by theta. In other words, one may reduce an upper bound on J(theta) without reducing the value of J(theta).

7) The Knill & Kersten example is a Directed Acyclic Graph (DAG). However, the neural network implementing this DAG has cycles (Fig 4C). What is the significance of this difference?

8) Does their approach work for undirected graphs? How would a neural model of an undirected cyclic graph (e.g. Markov Random Field) differ? If so, are the connection weights symmetric on average? If not, why not? If so, then the lack of symmetric that they highlight could just be due to stochasticity, which would have little practical relevance and in fact should impede good performance.

9) p5: The network learns joint probabilities of >x,z<. The examples provided on pages 3-4 suppose that x and z are both observable variables. Then, for recursive model construction, it is the observed output variables that are chained together. In this way, all intermediate (z) variables are actually fully observed, and it is only the alpha neurons (‘a’ variables) that are hidden. Although an arbitrary distribution can be constructed as a mixture distribution, just like the universal function approximator this construction is not a particularly parsimonious or efficient representation of complex structure. Thus this architecture seems like a serious constraint, that chained learning only works when many intermediate variables are observable. Probably deeper or more structured latent representations can be learned as well, but it is not clear to me that STDP will suffice for that.

10) What happens to the learning during spontaneous activity? I expect that random deviations from the true distribution will cause the learning to drift from the weights learned from the p* distribution over pinned inputs. Is there some factor to prevent this, e.g. that modulates plasticity to occur only during presentation of inputs?

11) p39: The importance of the ‘null’ values is unclear. How much representational benefit is there to adding this null value?

12) The authors seem to make a distinction between the internal model (which is a joint distribution) and what the neurons encode (which is a conditional distribution). This should be spelled out, and this symantic distinction should be justified: it seems like an awkward distinction to draw, because both joint and conditional distributions can be reasonably thought of as an internal model.

13) p19: “In fact, our model provides perhaps the first modelling framework where one can interpret on the level of the underlying network of neurons how such generally important priors could be implemented in the brain, and possibly even genetically encoded (or predisposed).” This is a dubious claim both for priority and content, and should be removed.

-------

Some more minor issues to correct:

awkward: “the shape of an EPSP varies between shapes with a pronounced initial peak and smooth hills in dependence of the distance of the synapse to the soma (Williams and Stuart, 2002), and receives yet other shapes if amplified through NMDA- or Ca- spikes” - The shape receives other shapes?

First full paragraph on p9 is awkward.

Fig 4 caption: "learning, module" should not have a comma.

"nominator", while apparently acceptable (news to me!), is highly nonstandard. "Numerator" is preferred.

p26: "the firing probability density of each neuron in alpha ideally remains constant" - I recommend adding "over time", since I first misinterpreted this sentence.

Figure 6A appears to have an error, as the cylindrical example has only the side wall on the left and the cap on the right.

it would have been helpful to specify earlier that the underlying model for one module is a mixture of multinomials.

On p19 it is stated that: "some type of feedforward inhibition that prevents hidden neurons for learning a probabilistic relationship between random variables (x,z) for a specific value z=l to engage plasticity for examples (x, z), with z not equaling l." How is this "some type of feedforward inhibition" implemented in the model? I think this is an awkward restatement of the imposed architectural constraint mentioned above.
